# Machine Learning–Based Survival Prediction Models for Young Patients With Gastric Cancer: Model Development and Validation Study

**DOI:** 10.2196/86418

**Published:** 2026-05-26

**Authors:** Ha Ye Jin Kang, Wooyeong Jang, Minsam Ko, Kwang Sun Ryu

**Affiliations:** 1 Department of Computer Science, Semyung University, Jecheon-si, Chungcheongbuk-do, Republic of Korea; 2 Department of Biomedical Engineering, School of Medicine, Chungbuk National University, Seowon-gu, Cheongju-si, Chungcheongbuk-do, Republic of Korea; 3 Department of Human-Computer Interaction, Hanyang University, Sangnok-gu, Ansan-si, Gyeonggi-do, Republic of Korea; 4 National Cancer Data Center, National Cancer Center, 323 Ilsan-ro, Ilsandong-gu, Goyang-si, Gyeonggi-do, 10408, Republic of Korea, 82 10-5769-7272, 82 10-031-920-0654

**Keywords:** survival prediction model, young patients with gastric cancer, machine learning, gastric cancer, mortality risk, predictive modeling

## Abstract

**Background:**

Despite a global decline in the incidence of gastric cancer (GC), the number of cases diagnosed among younger individuals continues to increase. Several studies have been conducted to develop predictive models of mortality in patients with GC.

**Objective:**

We developed 3- and 5-year survival prediction models for young patients with GC based on machine learning–based survival modeling approaches.

**Methods:**

Data from 813 young patients (≤50 years) diagnosed with GC between 2013 and 2015 were retrieved from the Gastric Cancer Public Staging Database. Among these 813 patients, data from 569 (70%) and 244 (30%) were allocated to the model training and testing datasets, respectively. Random survival forest, gradient boosting survival analysis, extra survival tree, and the Cox proportional hazards model were applied to predict survival outcomes at the 3- and 5-year time horizons. Model performance was assessed and quantified using the concordance index (C-index) metric. For the machine learning prediction models, the mean C-index values and corresponding 95% CIs were estimated across 100 repeated training iterations.

**Results:**

For the random survival forest model, the C-index for predicting 3-year mortality was 95.89% (95% CI 95.80%‐95.97%), whereas the C-index for predicting 5-year mortality was 91.82% (95% CI 91.68%‐91.96%). In the gradient boosting survival analysis model, the C-index for predicting 3-year mortality was 95.32% (95% CI 95.31%‐95.33%), and the C-index for predicting 5-year mortality was 89.98% (95% CI 89.95%‐90.01%). For the extra survival tree model, the C-index for predicting 3-year mortality was 95.53% (95% CI 95.46%‐95.60%), whereas the C-index for predicting 5-year mortality was 94.60% (95% CI 94.50%‐94.70%). In addition, the Cox proportional hazards model showed a C-index of 94.15% for predicting 3-year mortality and 82.26% for predicting 5-year mortality. Tumor stage and tumor size were the primary predictive variables used to train the models for mortality prediction at different time points. Other variables exhibited varying levels of predictive contribution across different time points.

**Conclusions:**

These findings may facilitate the identification of high-risk young patients with GC who may benefit from more aggressive treatment strategies by enabling the prediction of mortality risk at different time points.

## Introduction

Despite a consistent global decline in the incidence of gastric cancer (GC) resulting from improved understanding of the key etiological factors influencing its development, more than 1 million new cases are still diagnosed annually worldwide, and the disease remains associated with a high mortality rate [1]. Recent studies have shown that the prevalence of GC among younger individuals varies substantially across geographic regions and is increasing worldwide. This trend is particularly evident in Asian countries such as South Korea, Japan, and China [2-4] where GC occurs at a comparatively higher rate among younger populations [2]. Although the precise mechanisms underlying GC development in younger individuals remain unclear, both disease progression and prognosis are influenced by a complex interplay of factors, including pathological characteristics, molecular alterations, genomic features, dietary patterns, and lifestyle-related exposures. Consequently, several studies have been conducted to evaluate prognostic outcomes in younger patients with GC [5,6]. Previous studies predicting survival outcomes in young patients with GC have primarily relied on traditional statistical nomogram models. These models represent the relationships between clinical covariates and patient outcomes in an interpretable format and have demonstrated relatively stable predictive performance [7].

Recently, machine learning (ML)–based approaches have been explored as complementary tools to traditional prediction models [8,9], demonstrating particular strengths in capturing complex nonlinear relationships among predictor variables and analyzing large-scale, high-dimensional clinical datasets [10,11]. Several studies have demonstrated that ML-based survival models can provide meaningful prognostic insights for predicting time-to-event outcomes using structured clinical data collected in real-world health care settings [12-16]. However, in young patients with GC, baseline prognostic factors influencing survival change over time [17]. In addition, existing mortality prediction models typically provide predictions for a single time point, which may limit their ability to capture time-varying changes in prognostic factors that may occur in younger patients with GC [15]. Therefore, to account for the complex nature of GC prognosis, this study developed 3- and 5-year mortality prediction ML-based models for young patients with GC and constructed 3- and 5-year mortality prediction models to capture potential time-varying risk factors. This ML-based survival prediction study aimed to identify how key prognostic factors associated with mortality vary across different prediction horizons.

## Methods

### Study Design

**Figure 1.**
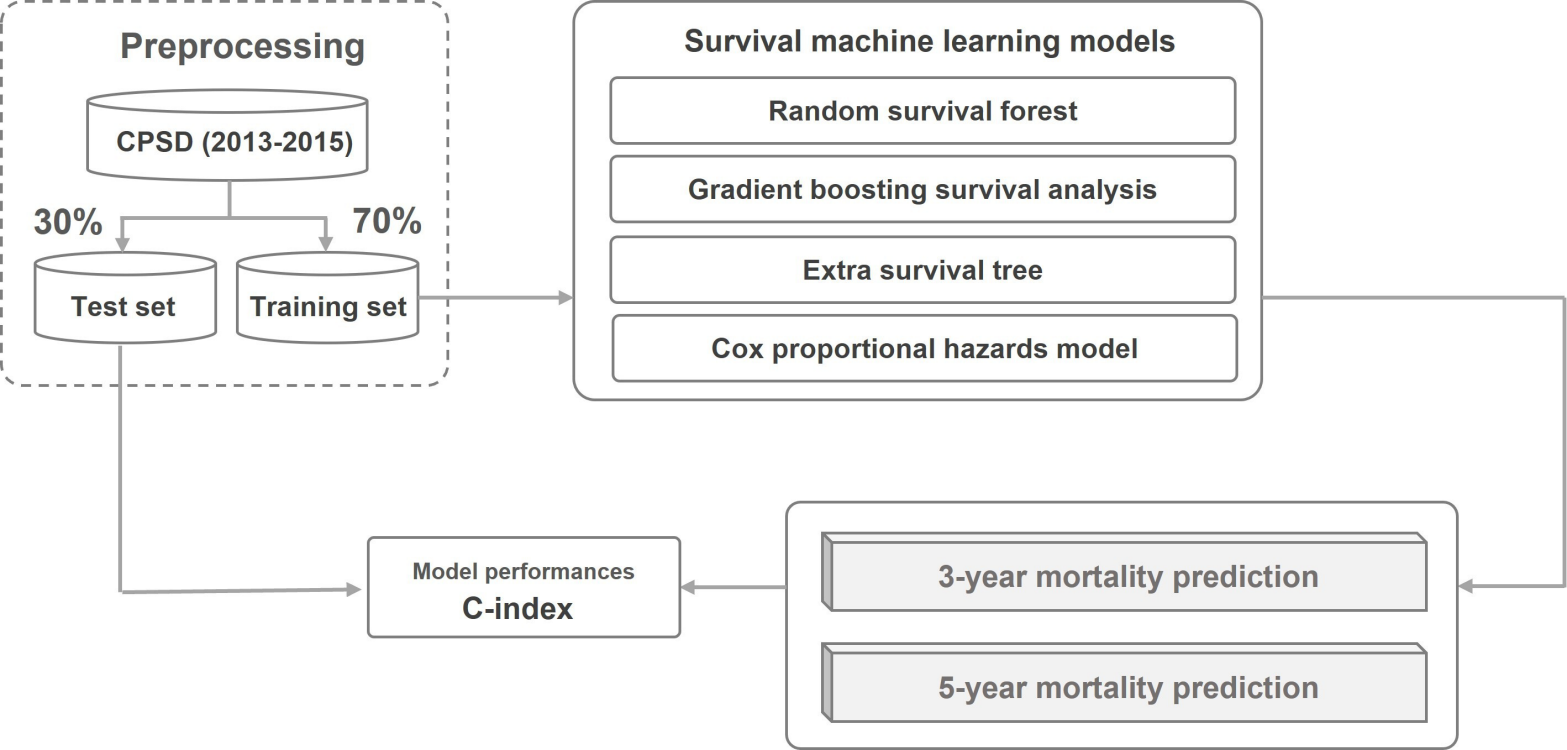
Research framework. CPSD: Cancer Public Staging Database.

We developed a predictive model to estimate all-cause mortality among younger patients with GC using deidentified data obtained from the Cancer Public Staging Database (CPSD) provided by the National Cancer Data Center (NCDC; Figure 1). To achieve this objective, we first extracted data for younger patients (≤50 years) diagnosed with GC between 2013 and 2015 from the CPSD and subsequently performed data preprocessing on the dataset. The data were randomly divided into training and testing subsets comprising 70% and 30% of the data, respectively. Using the training dataset, we developed 3- and 5-year mortality prediction models using random survival forest (RSF), gradient boosting survival analysis (GBSA), extra survival tree (EST), and Cox proportional hazards (PH).

### Ethical Considerations

This study was approved by the institutional review board of the National Cancer Center of South Korea (NCC2023-0260). The requirement for informed consent was waived because HYJK, WJ, and KSR accessed only anonymized data for the purposes of analysis. The pseudonymized dataset was analyzed within a secure research environment provided by the NCDC, ensuring that only aggregated analytical results were exported.

### Data Source

This study used the CPSD, which is provided by the NCDC. This database was constructed by linking data from the NCDC’s Cancer Public Library Database with collaborative staging data for cancer cases established in the Korea Central Cancer Registry (KCCR) [18]. The collaborative staging system was developed using probability-based sampling methods and mandatory retrospective medical record surveys of cancer cases registered in the KCCR. These surveys provide information on cancer staging, treatment modalities, and patient prognoses [19]. The Cancer Public Library Database was developed by integrating linked data from 4 major population-based public databases: the Korea National Cancer Incidence Database maintained by the KCCR, cause of death records from Statistics Korea, the National Health Information Database of the National Health Insurance Service, and the National Health Insurance Research Database maintained by the Health Insurance Review and Assessment Service [20]. The use of the CPSD dataset complied with the data access and use policies established by the NCDC, and all data were handled in accordance with relevant guidelines and regulations to ensure confidentiality and data security. As the dataset contained no personally identifiable information, this study was exempt from institutional review board review.

### Definitions

Comorbid conditions, including myocardial infarction, stroke, heart failure, diabetes mellitus, hypertension, dyslipidemia, chronic obstructive pulmonary disease, peripheral vascular disease, liver disease, atrial fibrillation, and chronic kidney disease, were identified and defined according to the diagnostic codes from the *International Classification of Diseases, 10th Revision*. Additionally, primary study end points were defined to represent short- and medium-term mortality, specifically all-cause mortality occurring within 3 and 5 years after cancer diagnosis, respectively. Information on all-cause mortality was obtained from official cause of death records provided by South Korea’s national statistical authority. Deaths occurring within each follow-up period were treated as cumulative mortality events; therefore, events occurring within 3 years were also included in the cumulative event count for the 5-year follow-up period.

### Experimental Dataset

The dataset was randomly divided into a training set (n=569) and a test set (n=244) using a fixed random seed (42) to ensure the reproducibility of the dataset partitioning. The primary outcome was all-cause mortality measured at 3 and 5 years after cancer diagnosis, and separate survival prediction models were developed for each prediction horizon. To minimize potential bias caused by outcome imbalance, the dataset was randomly partitioned using stratified sampling, ensuring comparable proportions of mortality events between the training and testing datasets. Model performance was evaluated on the corresponding test dataset using the Harrell C-index, a widely used discrimination metric in survival analysis. For the ML-based survival models (RSF, GBSA, and EST), the training and evaluation procedures were repeated across 100 iterations, with the random seed varied in each iteration while maintaining a fixed data split to quantify stochastic variability and assess the robustness of model performance. At each iteration, the models were refitted using the same training dataset and subsequently evaluated on the identical test dataset. The resulting training and testing datasets were fixed after partitioning and were consistently used in all subsequent model development and evaluation experiments.

### Models

The RSF is an ensemble-based ML method that models complex nonlinear relationships between predictor variables and estimates variable importance to reduce generalization error. The RSF model can also estimate the cumulative hazard function for individual observations even when the PH assumption is not satisfied [21,22]. A gradient boosting machine framework [23] was adapted for survival analysis, enabling the model to effectively handle light-censored survival data. Gradient boosting machine is an ensemble learning technique in which successive decision trees are sequentially constructed, with each tree iteratively correcting the prediction errors of the preceding model. GBSA builds on this boosting framework for survival modeling, allowing the algorithm to handle censored observations and capture complex interactions between predictor variables and survival time [24,25]. The EST model, an extension of the randomized trees algorithm introduced by Geurts et al [26] in 2006, introduces additional randomness by selecting split points randomly during tree construction. The EST algorithm can censor survival data and does not rely on the PH assumption [27]. The Cox PH model is a widely used semiparametric survival analysis method that examines the relationship between survival time and multiple predictor variables. This model does not require explicit specification of the baseline hazard function [28,29].

### Computational Environment Settings

Survival analysis is a statistical methodology used to analyze time-to-event outcomes such as death or disease progression [30], and ML approaches are increasingly being adopted in health care research to address the analytical complexities associated with survival modeling [31]. Nonparametric tree-based ensemble methods are generally considered well suited for analyzing censored time-to-event outcomes and performing dynamic survival predictions [32]. The predictor variables were further ranked and stratified according to their relative feature importance within the survival prediction models. In this study, we developed a survival prediction model based on GBSA, RSF, EST, and the Cox PH. During model development, feature importance scores were computed based on the mean decrease in impurity using the scikit-learn library (version 1.0.2; Google Summer of Code project) [33]. The models were implemented using the scikit-survival library (version 0.17.2) [34] within a Python (version 3.7.5; Python Software Foundation) computational environment, with TensorFlow (version 1.15.5; Google Brain Team) used as the underlying ML framework.

All survival models were trained using predefined and fixed hyperparameter configurations. For the RSF, GBSA, and EST models, the number of estimators was fixed at 200, whereas all remaining hyperparameters were retained at their default library settings. For the Cox PH model, elastic net regularization was applied with an L1 ratio of 1 × 10^–6^ and predefined alpha values to enhance numerical stability during model optimization. Continuous variables were not normalized for tree-based survival models because tree-based algorithms are inherently scale invariant and do not require feature normalization. Missing values were handled through data imputation using the median value for continuous variables and the most frequent category for categorical variables followed by one-hot encoding of categorical features. Importantly, all preprocessing procedures—including imputation and feature encoding—were fit exclusively on the training dataset and subsequently applied to the test dataset to prevent information leakage during model evaluation. For the Cox PH model, the predictor variables were standardized prior to model training to improve numerical stability and optimization convergence.

### Model Performance Evaluation

The C-index [35] is the most widely used evaluation metric for assessing the performance of survival prediction models [36]. This C-index is computed by comparing the predictive risk scores and observed survival times for pairs of randomly selected patients. To determine the number of concordant and discordant patient pairs, this pairwise comparison process was repeated across all possible patient pairs within the study cohort. The predictive model assigns a numerical risk score to each individual, representing the predicted risk of mortality, whereas the C-index evaluates the model’s ability to correctly rank the relative risk between pairs of individuals [37,38]. Therefore, the C-index was used to evaluate the predictive performance and discrimination ability of the survival prediction models.

## Results

### Study Population

**Figure 2.**
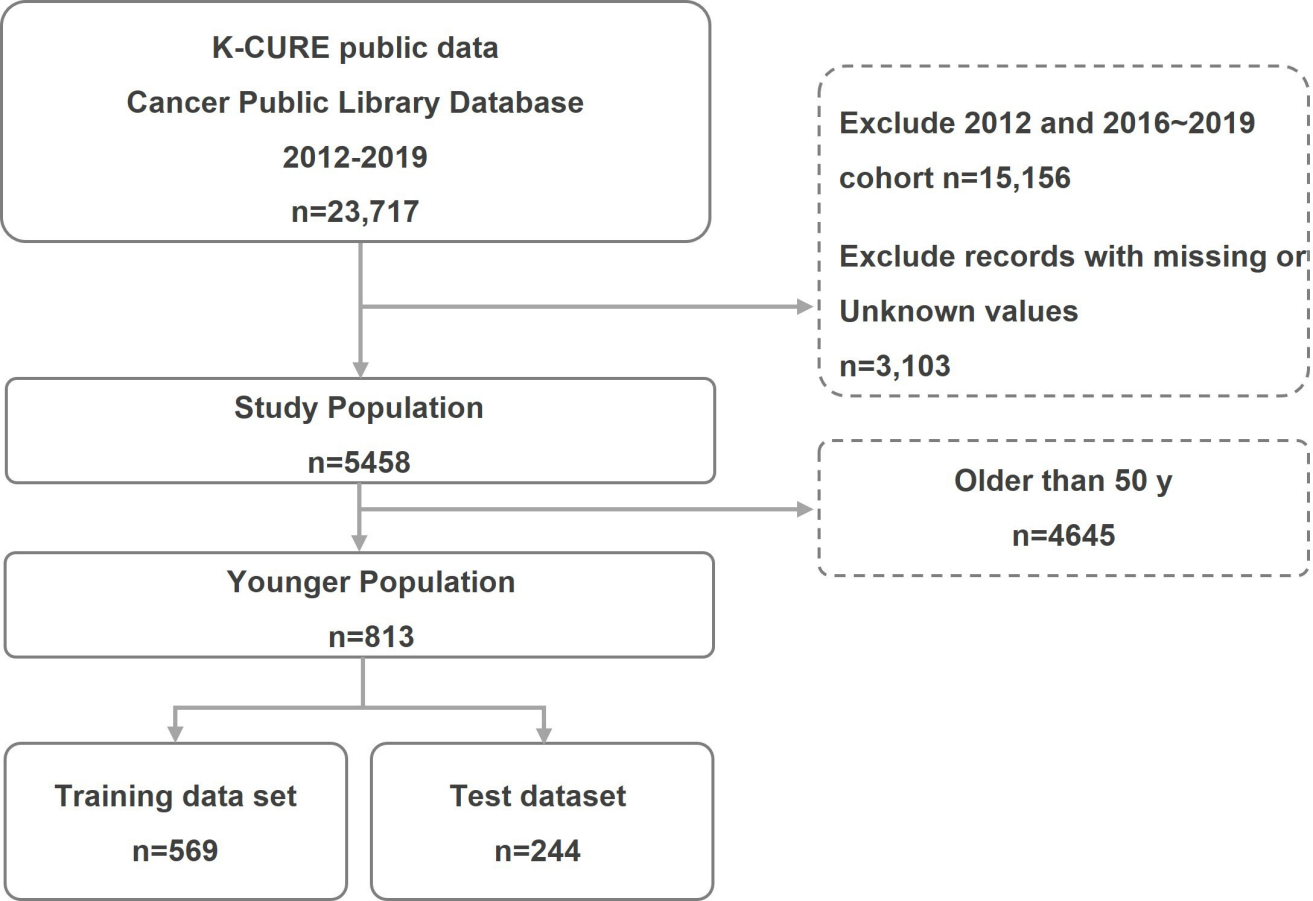
Generation process of the experimental datasets. K-CURE: Korea Clinical Data Utilization Network for Research Excellence.

The CPSD contains data for 23,717 individuals diagnosed with GC at the primary anatomical sites C160 to C166, C168, and C169 between 2012 and 2019. From this dataset, we excluded patients diagnosed with GC between 2016 and 2019 for 5-year mortality prediction analysis, as well as the 2012 cohort, for which prediagnostic screening information was unavailable. The dataset subsequently underwent data cleaning procedures, during which 3103 records containing missing or unknown values were removed, including tumor size (T-size; n=1140, 36.7%), height (n=1849, 59.6%), urine protein (n=44, 1.4%), gamma-glutamyl transpeptidase (n=4, 0.1%), low-density lipoprotein cholesterol (n=17, 0.5%), and estimated glomerular filtration rate (eGFR; n=49, 1.6%). Patients aged 50 years or younger at the time of diagnosis were classified as the “younger” patient group, as illustrated in Figure 2.

### Baseline Characteristics

The study population showed differences between younger and older patients. Among younger patients, there was a higher proportion of women, whereas older patients exhibited a higher burden of comorbidities, including cardiovascular and metabolic diseases. Differences were also observed in tumor-related characteristics, including cancer stage according to the seventh edition of the American Joint Committee on Cancer (AJCC) cancer staging manual, with younger patients tending to present with more advanced stages. In addition, older patients showed higher all-cause mortality at both 3 and 5 years. Detailed baseline characteristics are provided in Multimedia Appendix 1.

### Model Performance

The RSF, GBSA, EST, and Cox PH models were trained and evaluated to compare their predictive performance for survival outcomes at 2 time horizons (3 and 5 years). Table 1 presents the mean C-index values with corresponding 95% CIs, reflecting the predictive accuracy of the models in estimating mortality risk among young patients with GC at different prediction time points. For the ML prediction models, we reported the mean C-index and the corresponding empirical 95% CIs across 100 repeated training iterations, whereas the Cox PH model was evaluated using a single model run; therefore, CIs for the C-index were not estimated or reported. The C-index values obtained from each of the 100 training iterations for the prediction models are provided in Multimedia Appendix 2. For 3-year survival prediction, the RSF, GBSA, EST, and Cox PH models achieved C-index values of 95.89% (95% CI 95.80%‐95.97%), 95.32% (95% CI 95.31%‐95.33%), 95.53% (95% CI 95.46%‐95.60%), and 94.15%, respectively. For 5-year survival prediction, the corresponding C-index values were 91.82% (95% CI 91.68%‐91.96%), 89.98% (95% CI 89.95%‐90.01%), 94.60% (95% CI 94.50%‐94.70%), and 82.26%, as summarized in Table 1.

**Table 1.** Concordance index (C-index) with 95% CIs for survival prediction models and the Cox proportional hazards (PH) model.

Model	3 year, C-index (%; 95% CI)	5 year, C-index (%; 95% CI)
RSF^a^	95.89 (95.80-95.97)	91.82 (91.68-91.96)
GBSA^b^	95.32 (95.31-95.33)	89.98 (89.95-90.01)
EST^c^	95.53 (95.46-95.60)	94.60 (94.50-94.70)
Cox PH	94.15^d^	82.26

a RSF: random survival forest.

b GBSA: gradient boosting survival analysis.

c EST: extra survival tree.

d The C-index values reported for the machine learning models represent the mean values and corresponding 95% CIs obtained from 100 repeated model training iterations. The Cox PH model was evaluated using a single model run; therefore, CIs for the C-index were not estimated or reported.

For 3-year survival prediction, the RSF model identified several important predictors, including the AJCC stage, T-size, age, serum glutamic oxaloacetic transaminase (aspartate aminotransferase), eGFR, systolic blood pressure, serum glutamic pyruvic transaminase (aspartate aminotransferase), hemoglobin level, and tumor topography codes (C162 and C160). For 5-year survival prediction, the RSF model identified AJCC stage, T-size, deep vein thrombosis, age, tumor topography code (C163), gamma-glutamyl transpeptidase, tumor grade, diastolic blood pressure, hemoglobin level, and liver disease as key prognostic predictors. For 3-year survival prediction, the GBSA model identified several important predictors, including AJCC stage, hemoglobin level, tumor topography code (C169), T-size, fasting blood glucose, total cholesterol, weekly alcohol consumption, walking activity level, systolic blood pressure, and serum glutamic oxaloacetic transaminase. For 5-year survival prediction, the GBSA model identified the following important predictors: AJCC stage, T-size, tumor topography code (C169), weekly alcohol consumption, fasting blood glucose, hemoglobin level, diastolic blood pressure, serum glutamic pyruvic transaminase, tumor grade, and total cholesterol. For 3-year survival prediction, the EST model identified several important predictors, including AJCC stage, T-size, diabetes mellitus, tumor topography codes (C169 and C162), proteinuria, waist circumference, body weight, daily alcohol consumption, and serum glutamic oxaloacetic transaminase levels. For 5-year survival prediction, the most influential predictors identified by the EST model included AJCC stage, T-size, tumor topography code (C163), deep vein thrombosis, liver disease, diabetes mellitus, tumor grade, BMI, waist circumference, and height. For 3-year mortality prediction, the Cox PH model identified AJCC stage and T-size as the most influential predictors, followed by serum glutamic oxaloacetic transaminase, eGFR, triglycerides, age, high-density lipoprotein cholesterol, hemoglobin level, total cholesterol, and gamma-glutamyl transpeptidase. For 5-year mortality prediction, AJCC stage and T-size remained the dominant predictors, together with serum glutamic oxaloacetic transaminase, eGFR, age, high-density lipoprotein cholesterol, total cholesterol, hemoglobin level, triglycerides, and weekly alcohol consumption.

Feature importance analysis showed that AJCC stage and T-size were consistently the most influential predictors across all models and prediction time points, whereas other variables demonstrated model- and time-dependent variability. Detailed feature importance results are provided in Multimedia Appendix 3, the corresponding hazard ratio analyses are presented in Multimedia Appendix 4, and Multimedia Appendix 5 provides the mapping between heat map feature labels and the corresponding clinical variables reported in the manuscript tables.

## Discussion

### Principal Findings

In this study, we developed ML-based survival prediction models for young patients with GC to estimate 3- and 5-year mortality. Among the evaluated models, RSF and EST demonstrated superior predictive performance compared to the Cox PH model. Across all models and prediction horizons, AJCC stage and T-size consistently emerged as the most important predictors of mortality risk.

GC has traditionally been considered a disease predominantly affecting older adults; however, recent epidemiological studies have reported an increasing incidence among younger individuals [39-41]. These findings highlight the need for tailored prognostic models for younger patient populations. Our analysis results indicated that the older patient group exhibited a poorer prognosis than the younger patient group. Furthermore, the stage-specific survival curves demonstrated adverse poorer prognoses in the older patient cohort. However, for stage 3 and 4 disease, no statistically meaningful differences in survival prognosis were observed between the younger and older groups, as shown in Multimedia Appendix 6. Owing to age-related physiological and biological differences, treatment responses and survival outcomes may vary, which should be considered when developing survival prediction models [42,43]. Previous studies have reported promising results, demonstrating the potential of predictive modeling to generate meaningful insights into survival outcomes [5,6,44-48]. However, uncertainty remains regarding the consistency of model performance across different predictive time horizons in young patients with GC. The superior performance of ML-based models compared with the Cox PH model may be attributed to their ability to capture complex nonlinear relationships and interactions among clinical variables, which are not adequately addressed by traditional statistical approaches. These findings suggest that ML-based models may provide more accurate risk stratification, potentially supporting personalized treatment strategies and clinical decision-making for young patients with GC. In particular, the use of longitudinal clinical and comorbidity data in this study may have contributed to improved model performance, which is in line with previous studies exploring the use of ML approaches for survival prediction. Furthermore, although many predictive models have been developed using the Surveillance, Epidemiology, and End Results database, which contains comprehensive cancer registry information, there remains limited availability of longitudinal data for tracking comorbidities and patient health characteristics, such as disease diagnosis codes (*International Classification of Diseases, 10th Revision*), medication records, and routine health examination data. Such information is not consistently available in the Healthcare Examiners and Evaluators database or the National Health Service database, which may influence the predictive performance and reliability of ML models. To address these limitations, we classified patients with GC as younger (≤50 years) based on criteria used in recent studies [49] and evaluated their 3- and 5-year survival predictions using multiple survival prediction models. Using feature importance analysis, we identified clinically relevant predictors, among which 2 variables consistently influenced survival prognosis across all models and time horizons: AJCC stage and T-size. These variables demonstrated significant associations with all-cause mortality at both the 3- and 5-year follow-up intervals, as shown in Multimedia Appendix 7. The tumor, node, metastasis (TNM) staging system represents a comprehensive framework for evaluating the anatomical extent of cancer progression and continues to serve as the fundamental basis for prognostic stratification in oncology. Although T-size contributes to the T component of the TNM classification, the continuous measurement of T-size may provide additional prognostic granularity beyond categorical stage classifications. The TNM staging system aggregates information from the tumor, nodal involvement, and metastasis categories into discrete stage groups. However, a continuous T-size variable may capture heterogeneity within the same stage group, which may not be fully represented by categorical staging alone. This may prove why both tumor stage and T-size were retained as significant predictors in the ML survival prediction models. These findings suggest that, within established staging frameworks, incorporating quantitative tumor characteristics such as continuous T-size measurements can provide additional prognostic information, particularly for long-term survival prediction. However, as illustrated by the heat map of key predictors for the 3- and 5-year models, the relative importance of variables used to predict mortality varied across prediction time points in Multimedia Appendix 3. The proposed survival prediction models enabled the identification of high-risk subgroups among young patients with GC by accounting for temporal variations in prognostic risk factors. These findings may support the development of more targeted and potentially aggressive treatment strategies for young patients with GC. To evaluate the performance of the proposed model, a comparative analysis was conducted between the traditional TNM staging system and the ML survival model for 1-, 3-, and 5-year mortality prediction, with the Harrell C-index used to quantify model discrimination performance. The findings demonstrated that ML-based approaches exhibited superior performance for 3- and 5-year mortality, whereas at the 1-year prediction horizon, the TNM staging classification showed slightly higher discriminatory power than the ML survival model, as shown in Multimedia Appendix 8. Consequently, the ML-based 1-year mortality prediction model was excluded from subsequent analyses. These findings suggest that short-term mortality outcomes are predominantly influenced by the initial stage of disease, a factor that is effectively captured by the conventional TNM staging classification system. Conversely, the additional predictive benefit of ML-based survival models became more pronounced in long-term predictions (3 and 5 years), where multiple complex clinical and biological factors may contribute to heterogeneity in patient outcomes. The validation of predictive model performance was strongly influenced by the data-splitting strategy used for model training and evaluation. This study compared the results obtained using a random data-splitting strategy (training: 70%; testing: 30%) applied to the entire dataset from 2013 to 2015 with those obtained using a year-based split (training: 2013‐2014; testing: 2015), as shown in Multimedia Appendix 9. The analysis demonstrated that the random data-splitting strategy produced a higher predictive performance compared with the year-based splitting approach. This result was interpreted as reflecting the inability of the year-based split to adequately represent the characteristics of the patient cohort collected in a specific year (2015) within the training dataset, which led to larger differences in key variables—particularly AJCC stage—between the training and testing datasets. In summary, although the dataset in this study was longitudinal and incorporated patient-specific follow-up durations, the year-based segmentation approach functioned more as a method for separating patient cohorts collected at different time points than as a validation framework representing a true temporal prediction scenario. This heterogeneity in key predictor variables—particularly AJCC stage—between the training and test datasets was considered to limit the generalization of latent patterns learned by the models during training, thereby reducing the overall predictive performance.

This study has several limitations. It was conducted using data derived from a single ethnic population, which may have introduced potential population-specific bias into the developed prediction models. In addition, external validation using independent datasets was not performed, which may limit the robustness, generalizability, and clinical applicability of these predictive models. Although this study developed and validated ML models for predicting 3- and 5-year mortality in young patients with GC, several important methodological considerations, including model interpretability and robustness to complex or incomplete data, were not fully addressed.

In summary, this study developed and validated ML models for predicting 3- and 5-year mortality in young patients with GC, demonstrating their potential utility for risk stratification and clinical decision-making. Validation using larger and more diverse patient populations is necessary to ensure robustness, external validity, and generalizability. The integration of explainable artificial intelligence methodologies may enhance the interpretability and transparency of ML models, thereby making predictive outputs more clinically actionable for health care practitioners [50]. Recent ML research has addressed several methodological challenges, including modeling temporal dependencies, discriminative feature selection, and improving robustness to noisy or incomplete input data. Prior studies in nonclinical ML domains have proposed advanced methodologies, including attention-based temporal models, structure-aware feature selection frameworks, and confidence-driven learning strategies [51-53]. Although these approaches do not directly fall within the scope of this study, they provide conceptually important insights for survival prediction problems, particularly those requiring careful handling of longitudinal data patterns, high-dimensional clinical variables, and uncertainty in medical datasets. These methodological directions may be adapted and extended to medical prognostic prediction models, particularly in survival analysis and clinical outcome prediction research. In addition to these methodological considerations, evaluation of the clinical utility of the key prognostic variables identified in this study, including cancer stage and T-size, is required through prospective or independent validation studies, whereas the use of internationally standardized and multiethnic datasets may further improve the reliability, external validity, and global generalizability of the predictive models.

### Conclusions

The number of young patients diagnosed with GC continues to increase worldwide, and nomogram-based prognostic models have been proposed to identify patients at high risk of adverse outcomes. However, relatively few studies have focused on predicting survival outcomes in young patients with GC using ML approaches. Therefore, we developed ML-based prediction models for estimating 3- and 5-year mortality in young patients with GC. These models may assist clinicians in identifying young patients with GC who may require more aggressive or personalized treatment strategies. However, because this analysis was conducted using data derived from the Korean patient population, future studies should externally validate these prediction models in diverse international populations.

## Supplementary material

10.2196/86418 Multimedia Appendix 1 Baseline characteristics of the older and younger patient groups.

10.2196/86418 Multimedia Appendix 2 C-index values of the survival prediction models.

10.2196/86418 Multimedia Appendix 3 Relative feature importance of predictors in the survival prediction model.

10.2196/86418 Multimedia Appendix 4 Univariate analysis results for all causes of death.

10.2196/86418 Multimedia Appendix 5 Mapping between heat map feature labels and clinical variables.

10.2196/86418 Multimedia Appendix 6 Survival curve for younger and older patients.

10.2196/86418 Multimedia Appendix 7 Survival curve for stage and tumor size.

10.2196/86418 Multimedia Appendix 8 Comparison of predictive performance between American Joint Committee on Cancer stage and survival machine learning models.

10.2196/86418 Multimedia Appendix 9 Comparison of year-based data splitting and the 7:3 random split.

## Abbreviations

AJCC: American Joint Committee on Cancer
CPSD: Cancer Public Staging Database
eGFR: estimated glomerular filtration rate
EST: extra survival tree
GBSA: gradient boosting survival analysis
GC: gastric cancer
KCCR: Korea Central Cancer Registry
ML: machine learning
NCDC: National Cancer Data Center
PH: proportional hazards
RSF: random survival forest
TNM: tumor, node, metastasis
