# Supplementary material for: Machine Learning–Based Survival Prediction Models for Young Patients With Gastric Cancer: Model Development and Validation Study
Source: JMIR Cancer. 2026 May 26;12:e86418. doi: 10.2196/86418 (PMC13211600; doi:10.2196/86418)
Supplement: Multimedia Appendix 1 [file cancer-v12-e86418-s001.docx]

**Supplement file 1. Baseline characteristic of the older and younger patient groups**

The baseline demographic and clinical characteristics of the younger and older patient groups were compared and summarized.

| **Variables** | **OlderGroup**  **N = 4,645** | | **Younger Group**  **N=813** | |
| --- | --- | --- | --- | --- |
|  | Mean±std | Median | Mean±std | Median |
| Age (year) | 65.36±8.28 | 66 | 44.29±4.73 | 45 |
| Female (%) | 28.55% | - | 40.96% | - |
| BMI (kg/㎡) | 23.59±2.7 | 23 | 23.33±2.99 | 23 |
| Height (cm) |  |  |  |  |
| 150 ≤ H <160 | 32.9% | - | 24.6% | - |
| 160 ≤ H <170 | 46.16% | - | 37.02% | - |
| 170 ≤ H < 180 | 20.02% | - | 33.21% | - |
| 180 ≤ H | 0.93% | - | 5.17% | - |
| Weight (kg, %) |  |  |  |  |
| 40 ≤ W < 50 | 8.53% | - | 8% | - |
| 50 ≤ W < 60 | 28.33% | - | 27.18% | - |
| 60 ≤ W < 70 | 35.84% | - | 28.29% | - |
| 70 ≤ W < 80 | 20.93% | - | 23.37% | - |
| 80 ≤ W < 90 | 5.45% | - | 8.49% | - |
| 90 ≤ W | 0.93% | - | 4.67% | - |
| Waist circumference (cm, %) |  |  |  |  |
| 60 ≤ WC < 70 | 4.65% | - | 16.24% | - |
| 70 ≤ WC < 90 | 26.05% | - | 33.58% | - |
| 80 ≤ WC < 90 | 45.06% | - | 32.84% | - |
| 90 ≤ WC | 24.24% | - | 17.34% | - |
| Systolic blood pressure (mmHg) | 127.29±14.93 | 128 | 119.16±13.73 | 120 |
| Diastolic blood pressure (mmHg) | 77.21±9.64 | 78 | 75.12±9.99 | 74 |
| Haemoglobin level (g/dL) | 13.79±1.80 | 14 | 14.00±1.96 | 14.1 |
| Fasting blood glucose (mg/dL) | 106.17±29.69 | 99 | 98.48±21.99 | 94 |
| Total cholesterol (mg/dL) | 189.13±39.00 | 113 | 192.45±34.80 | 197 |
| Serum glutamic oxaloacetic transaminase (IU/L) | 27.79±18.47 | 24 | 25.39±14.45 | 22 |
| Serum glutamic pyruvic transaminase (IU/L) | 24.62±17.80 | 20 | 25.66±20.67 | 20 |
| Gamma glutamyl transpeptidase (IU/L) | 42.32±61.12 | 26 | 43.48±64.34 | 24 |
| Triglycerides (mg/dL) | 134.69±84.52 | 113 | 136.60±103.34 | 109 |
| High-density lipoprotein (mg/dL) | 51.89±19.28 | 50 | 53.30±14.23 | 51 |
| Low-density lipoprotein (mg/dL) | 110.86±35.00 | 109 | 112.98±37.45 | 111 |
| Serum creatine (mg/dL) | 0.94±0.43 | 0.9 | 0.88±0.35 | 0.9 |
| Estimated glomerular filtration rate (mL/min) | 84.20±25.44 | 81 | 91.97±19.69 | 89 |
| Protein in urine |  |  |  |  |
| 1 negative (-) | 92.98% | - | 93.6% | - |
| 2 positive (±) | 3.12% | - | 4.18% | - |
| 3 positive (+1) | 2.43% | - | 1.35% | - |
| 4 positive (+2) | 1.16% | - | *** | - |
| 5 positive (+3) | 0.28% | - | *** | - |
| 6 positive (+4) | *** | - | *** | - |
| TopographyCODE (%) |  |  |  |  |
| C160 | 3.98% | - | 3.44% | - |
| C161 | 0.6% | - | 0.98% | - |
| C162 | 35.52% | - | 50.92% | - |
| C163 | 50.12% | - | 36.53% | - |
| C164 | 1.4% | - | 0.74% | - |
| C165 | 1.61% | - | 1.72% | - |
| C166 | *** | - | *** | - |
| C168 | 3.21% | - | 2.34% | - |
| C169 | 3.51% | - | 3.32% | - |
| Morphology CODE (%) |  |  |  |  |
| 1. Squamous and transitional cell carcinoma (8051– 8084, 8120–8131)) | 0.28% | - | *** | - |
| 3. Adenocarcinoma (8140–8149, 8160–8163, 8190–8221, 8260–8337, 8350–8552, 8570–8576, 8940–8941) | 98.73% | - | 98.89% | - |
| 4. Other specific carcinomas (8030–8046, 8150–8157, 8170–8180, 8230–8255, 8340–8347, 8560–8562, 8580–8671) | 0.22% | - | *** | - |
| 5. Unspecified carcinomas (NOS) (8010–8015, 8020–8022, 8050) | 0.71% | - | 0.98% | - |
| 17. Unspecified types of cancer (8000–8005) | *** | - | *** | - |
| GRADE (%) |  |  |  |  |
| 1.Well-differentiated, differentiated, NOS | 29.75% | - | 11.81% | - |
| 2.Moderately differentiated, moderately well differentiated, intermediate differentiation | 34.12% | - | 19.8% | - |
| 3.Poorly differentiated; dedifferentiated | 24.39% | - | 36.65% | - |
| 4.Undifferentiated, anaplastic | *** | - | *** | - |
| 9.Differentiation unknown, not stated, or not applicable | 11.71% | - | 31.73% | - |
| AJCC7 STAGE (%) |  |  |  |  |
| IA | 71.15% | - | 68.63 | - |
| IB | 7.06% | - | 6.52 | - |
| IIA | 4.56% | - | 5.66 | - |
| IIB | 4.24% | - | 4.43 | - |
| IIIA | 2.69% | - | 3.57 | - |
| IIIB | 2.97% | - | 2.95 | - |
| IIIC | 3.9% | - | 4.06 | - |
| IIIN | *** | - | *** | - |
| IV | 3.12% | - | 3.81 | - |
| Unknown | 0.28% | - | *** | - |
| T-size | 29.74±26.69 | 22 | 31.38±28.74 | 22 |
| Atrial fibrillation (%) | 7.38% |  | 1.85 |  |
| Chronic kidney disease (%) | 6.24% |  | 1.6 |  |
| Chronic obstructive pulmonary disease (%) | 25.96% |  | 10.09 |  |
| Diabetes (%) | 69.24% |  | 44.77 |  |
| Deep vein thrombosis (%) | 6.33% |  | 3.08 |  |
| Dyslipidaemia (%) | 88.03% |  | 77.61 |  |
| Heart failure (%) | 20.5% |  | 6.15 |  |
| Hypertension (%) | 67.34% |  | 25.71 |  |
| Liver disease (%) | 78.11% |  | 70.36 |  |
| Myocardial infarction (%) | 7.13% |  | 1.6 |  |
| Obesity(%) | 0.13% |  | *** |  |
| Stroke (%) | 2.95% |  | 0.86 |  |
| Smoking status (%) |  |  |  |  |
| Non-smoker | 48.01% |  | 48.95 |  |
| Past smoker | 28.53% |  | 17.71 |  |
| Current smoker | 23.47% |  | 33.33 |  |
| Weekly alcohol consumption (days) | 1.21±1.90 | 0 | 1.28±1.50 | 1 |
| Daily alcohol consumption (glasses) | 2.34±3.67 | 0 | 3.83±4.52 | 2 |
| Vigorous physical activity  (days in a week) | 1.04±1.85 | 0 | 1.05±1.61 | 0 |
| Moderate physical activity  (days in a week) | 1.34±2.08 | 0 | 1.25±1.69 | 0 |
| Physical activity Walking  (days in a week) | 2.98±2.66 | 3 | 2.63±2.37 | 2 |
| 3 year survival time (days) | 1,028.62±215.25 | 1,096 | 1,055.87±161.84 | 1,096 |
| 5 year survival time (days) | 1659.89±435.42 | 1,826 | 1,720.3±347.57 | 1,826 |
| All cause of death for 3 year (%) | 11.13% |  | 7.26% |  |
| All cause of death for 5 year (%) | 15.69% |  | 9.96% |  |

*** count less than or equal to 5
