# Supplementary material for: Machine Learning–Based Survival Prediction Models for Young Patients With Gastric Cancer: Model Development and Validation Study
Source: JMIR Cancer. 2026 May 26;12:e86418. doi: 10.2196/86418 (PMC13211600; doi:10.2196/86418)
Supplement: Multimedia Appendix 2 [file cancer-v12-e86418-s002.docx]

**Supplement file 2. C-Index values of survival prediction models**

The tables present the C-Index (concordance index) values of survival prediction models (RSF, GBSA, EST, COXPH) across 100 iterations for young patients with gastric cancer at each time point: 1 year, 3 years, and 5 years.

| **Model** | **Seed** | **3-year** | **5-year** |
| --- | --- | --- | --- |
| **RSF** | 1 | 0.960657 | 0.914838 |
|  | 2 | 0.951872 | 0.907785 |
|  | 3 | 0.956073 | 0.902038 |
|  | 4 | 0.962567 | 0.919018 |
|  | 5 | 0.958747 | 0.911964 |
|  | 6 | 0.959129 | 0.904389 |
|  | 7 | 0.965623 | 0.918495 |
|  | 8 | 0.964477 | 0.906479 |
|  | 9 | 0.961803 | 0.908568 |
|  | 10 | 0.957983 | 0.930251 |
|  | 11 | 0.956073 | 0.917712 |
|  | 12 | 0.959511 | 0.92581 |
|  | 13 | 0.962185 | 0.912226 |
|  | 14 | 0.961039 | 0.912748 |
|  | 15 | 0.965623 | 0.91954 |
|  | 16 | 0.957601 | 0.932602 |
|  | 17 | 0.962949 | 0.909613 |
|  | 18 | 0.954545 | 0.922936 |
|  | 19 | 0.952254 | 0.918495 |
|  | 20 | 0.963713 | 0.910397 |
|  | 21 | 0.961421 | 0.92999 |
|  | 22 | 0.963331 | 0.914577 |
|  | 23 | 0.961803 | 0.920846 |
|  | 24 | 0.959129 | 0.91745 |
|  | 25 | 0.961039 | 0.900993 |
|  | 26 | 0.957601 | 0.928422 |
|  | 27 | 0.959129 | 0.913009 |
|  | 28 | 0.963331 | 0.923981 |
|  | 29 | 0.964477 | 0.927377 |
|  | 30 | 0.958747 | 0.915883 |
|  | 31 | 0.964859 | 0.916667 |
|  | 32 | 0.960275 | 0.920063 |
|  | 33 | 0.956073 | 0.91745 |
|  | 34 | 0.954545 | 0.927638 |
|  | 35 | 0.961803 | 0.920846 |
|  | 36 | 0.958365 | 0.92581 |
|  | 37 | 0.964859 | 0.922414 |
|  | 38 | 0.962185 | 0.90883 |
|  | 39 | 0.956837 | 0.909613 |
|  | 40 | 0.954545 | 0.925287 |
|  | 41 | 0.957219 | 0.916928 |
|  | 42 | 0.962185 | 0.913793 |
|  | 43 | 0.959511 | 0.937827 |
|  | 44 | 0.953782 | 0.916144 |
|  | 45 | 0.956073 | 0.916667 |
|  | 46 | 0.961421 | 0.918757 |
|  | 47 | 0.954927 | 0.921369 |
|  | 48 | 0.960657 | 0.914838 |
|  | 49 | 0.966387 | 0.91954 |
|  | 50 | 0.961421 | 0.921108 |
|  | 51 | 0.952636 | 0.923459 |
|  | 52 | 0.963713 | 0.924765 |
|  | 53 | 0.964477 | 0.920063 |
|  | 54 | 0.953018 | 0.911181 |
|  | 55 | 0.958365 | 0.922414 |
|  | 56 | 0.961421 | 0.909352 |
|  | 57 | 0.953018 | 0.915361 |
|  | 58 | 0.955691 | 0.927377 |
|  | 59 | 0.957219 | 0.910397 |
|  | 60 | 0.956455 | 0.913793 |
|  | 61 | 0.955691 | 0.918757 |
|  | 62 | 0.955309 | 0.926594 |
|  | 63 | 0.964095 | 0.924765 |
|  | 64 | 0.959893 | 0.915622 |
|  | 65 | 0.950726 | 0.917712 |
|  | 66 | 0.961421 | 0.922414 |
|  | 67 | 0.960657 | 0.91954 |
|  | 68 | 0.959129 | 0.915622 |
|  | 69 | 0.962567 | 0.921891 |
|  | 70 | 0.950344 | 0.916405 |
|  | 71 | 0.956837 | 0.916144 |
|  | 72 | 0.963331 | 0.917973 |
|  | 73 | 0.959511 | 0.919018 |
|  | 74 | 0.956837 | 0.935214 |
|  | 75 | 0.959511 | 0.916144 |
|  | 76 | 0.956455 | 0.914316 |
|  | 77 | 0.959893 | 0.917712 |
|  | 78 | 0.955691 | 0.921108 |
|  | 79 | 0.960657 | 0.915361 |
|  | 80 | 0.947288 | 0.919279 |
|  | 81 | 0.958747 | 0.921108 |
|  | 82 | 0.956073 | 0.922153 |
|  | 83 | 0.958747 | 0.928945 |
|  | 84 | 0.956837 | 0.902038 |
|  | 85 | 0.960657 | 0.909875 |
|  | 86 | 0.957983 | 0.912748 |
|  | 87 | 0.957601 | 0.909352 |
|  | 88 | 0.94385 | 0.920846 |
|  | 89 | 0.959129 | 0.916405 |
|  | 90 | 0.965241 | 0.922936 |
|  | 91 | 0.960657 | 0.937043 |
|  | 92 | 0.955691 | 0.907262 |
|  | 93 | 0.964095 | 0.91745 |
|  | 94 | 0.959129 | 0.912487 |
|  | 95 | 0.959129 | 0.920324 |
|  | 96 | 0.964859 | 0.918234 |
|  | 97 | 0.957983 | 0.912748 |
|  | 98 | 0.959129 | 0.921369 |
|  | 99 | 0.95149 | 0.928422 |
|  | 100 | 0.958365 | 0.920063 |
| **GBSA** | 1 | 0.953782 | 0.900209 |
|  | 2 | 0.9534 | 0.901515 |
|  | 3 | 0.953018 | 0.90047 |
|  | 4 | 0.952636 | 0.90034 |
|  | 5 | 0.952636 | 0.901385 |
|  | 6 | 0.953018 | 0.900993 |
|  | 7 | 0.953782 | 0.900862 |
|  | 8 | 0.9534 | 0.90047 |
|  | 9 | 0.9534 | 0.898119 |
|  | 10 | 0.9534 | 0.902168 |
|  | 11 | 0.9534 | 0.900731 |
|  | 12 | 0.9534 | 0.89838 |
|  | 13 | 0.9534 | 0.900731 |
|  | 14 | 0.954163 | 0.90047 |
|  | 15 | 0.9534 | 0.898642 |
|  | 16 | 0.953782 | 0.901776 |
|  | 17 | 0.9534 | 0.900601 |
|  | 18 | 0.9534 | 0.900993 |
|  | 19 | 0.9534 | 0.901515 |
|  | 20 | 0.952636 | 0.898642 |
|  | 21 | 0.952636 | 0.90047 |
|  | 22 | 0.953782 | 0.900993 |
|  | 23 | 0.953018 | 0.898119 |
|  | 24 | 0.9534 | 0.900993 |
|  | 25 | 0.9534 | 0.900731 |
|  | 26 | 0.9534 | 0.900993 |
|  | 27 | 0.9534 | 0.897858 |
|  | 28 | 0.953782 | 0.898903 |
|  | 29 | 0.952254 | 0.901123 |
|  | 30 | 0.953018 | 0.897858 |
|  | 31 | 0.9534 | 0.898642 |
|  | 32 | 0.953782 | 0.897597 |
|  | 33 | 0.953018 | 0.899948 |
|  | 34 | 0.9534 | 0.901515 |
|  | 35 | 0.9534 | 0.898119 |
|  | 36 | 0.9534 | 0.901254 |
|  | 37 | 0.953018 | 0.898119 |
|  | 38 | 0.9534 | 0.89838 |
|  | 39 | 0.953018 | 0.900731 |
|  | 40 | 0.9534 | 0.90034 |
|  | 41 | 0.954163 | 0.898119 |
|  | 42 | 0.952254 | 0.89838 |
|  | 43 | 0.9534 | 0.897858 |
|  | 44 | 0.952636 | 0.897858 |
|  | 45 | 0.9534 | 0.900731 |
|  | 46 | 0.9534 | 0.898119 |
|  | 47 | 0.953018 | 0.901907 |
|  | 48 | 0.952636 | 0.897858 |
|  | 49 | 0.9534 | 0.900993 |
|  | 50 | 0.9534 | 0.900731 |
|  | 51 | 0.9534 | 0.898119 |
|  | 52 | 0.9534 | 0.900209 |
|  | 53 | 0.952636 | 0.899948 |
|  | 54 | 0.9534 | 0.898119 |
|  | 55 | 0.9534 | 0.899687 |
|  | 56 | 0.952636 | 0.900993 |
|  | 57 | 0.9534 | 0.901254 |
|  | 58 | 0.952636 | 0.901123 |
|  | 59 | 0.9534 | 0.900993 |
|  | 60 | 0.953018 | 0.901123 |
|  | 61 | 0.9534 | 0.898119 |
|  | 62 | 0.953018 | 0.900993 |
|  | 63 | 0.9534 | 0.901907 |
|  | 64 | 0.953782 | 0.901646 |
|  | 65 | 0.9534 | 0.900731 |
|  | 66 | 0.952636 | 0.900209 |
|  | 67 | 0.9534 | 0.898642 |
|  | 68 | 0.952636 | 0.898119 |
|  | 69 | 0.9534 | 0.898903 |
|  | 70 | 0.9534 | 0.897858 |
|  | 71 | 0.9534 | 0.898119 |
|  | 72 | 0.9534 | 0.900993 |
|  | 73 | 0.953782 | 0.90047 |
|  | 74 | 0.9534 | 0.900209 |
|  | 75 | 0.952636 | 0.901385 |
|  | 76 | 0.9534 | 0.900731 |
|  | 77 | 0.952636 | 0.898903 |
|  | 78 | 0.953782 | 0.897858 |
|  | 79 | 0.953782 | 0.901254 |
|  | 80 | 0.952636 | 0.898119 |
|  | 81 | 0.952636 | 0.901254 |
|  | 82 | 0.952636 | 0.900862 |
|  | 83 | 0.9534 | 0.90047 |
|  | 84 | 0.9534 | 0.899556 |
|  | 85 | 0.952636 | 0.89838 |
|  | 86 | 0.9534 | 0.897858 |
|  | 87 | 0.953018 | 0.900993 |
|  | 88 | 0.9534 | 0.898119 |
|  | 89 | 0.952636 | 0.90034 |
|  | 90 | 0.953018 | 0.901123 |
|  | 91 | 0.9534 | 0.898119 |
|  | 92 | 0.953782 | 0.897335 |
|  | 93 | 0.953018 | 0.901646 |
|  | 94 | 0.952636 | 0.89838 |
|  | 95 | 0.953782 | 0.89838 |
|  | 96 | 0.953018 | 0.89838 |
|  | 97 | 0.952636 | 0.901254 |
|  | 98 | 0.9534 | 0.901123 |
|  | 99 | 0.9534 | 0.900731 |
|  | 100 | 0.9534 | 0.898903 |
| **EST** | 1 | 0.959129 | 0.939133 |
|  | 2 | 0.961421 | 0.944096 |
|  | 3 | 0.957219 | 0.94488 |
|  | 4 | 0.950344 | 0.942529 |
|  | 5 | 0.954545 | 0.952978 |
|  | 6 | 0.955691 | 0.942529 |
|  | 7 | 0.954163 | 0.946447 |
|  | 8 | 0.953782 | 0.949321 |
|  | 9 | 0.9534 | 0.946186 |
|  | 10 | 0.954545 | 0.947753 |
|  | 11 | 0.964095 | 0.952978 |
|  | 12 | 0.949962 | 0.950104 |
|  | 13 | 0.958747 | 0.952717 |
|  | 14 | 0.951872 | 0.953239 |
|  | 15 | 0.948816 | 0.944619 |
|  | 16 | 0.956455 | 0.945925 |
|  | 17 | 0.956455 | 0.940961 |
|  | 18 | 0.953018 | 0.949321 |
|  | 19 | 0.957219 | 0.951411 |
|  | 20 | 0.955309 | 0.945402 |
|  | 21 | 0.957601 | 0.947231 |
|  | 22 | 0.953782 | 0.940439 |
|  | 23 | 0.952636 | 0.945664 |
|  | 24 | 0.959511 | 0.948015 |
|  | 25 | 0.962567 | 0.946186 |
|  | 26 | 0.954163 | 0.952194 |
|  | 27 | 0.957219 | 0.948537 |
|  | 28 | 0.950726 | 0.947492 |
|  | 29 | 0.958365 | 0.939133 |
|  | 30 | 0.960657 | 0.947492 |
|  | 31 | 0.952636 | 0.950888 |
|  | 32 | 0.954927 | 0.945664 |
|  | 33 | 0.956073 | 0.935737 |
|  | 34 | 0.957601 | 0.94279 |
|  | 35 | 0.952636 | 0.940961 |
|  | 36 | 0.958365 | 0.942529 |
|  | 37 | 0.956073 | 0.943051 |
|  | 38 | 0.956073 | 0.952194 |
|  | 39 | 0.956455 | 0.950366 |
|  | 40 | 0.959893 | 0.944357 |
|  | 41 | 0.958365 | 0.930512 |
|  | 42 | 0.952636 | 0.944619 |
|  | 43 | 0.953782 | 0.948276 |
|  | 44 | 0.953018 | 0.939133 |
|  | 45 | 0.959893 | 0.939655 |
|  | 46 | 0.952636 | 0.948015 |
|  | 47 | 0.954927 | 0.946447 |
|  | 48 | 0.957983 | 0.939133 |
|  | 49 | 0.949962 | 0.946186 |
|  | 50 | 0.954545 | 0.946708 |
|  | 51 | 0.957983 | 0.952456 |
|  | 52 | 0.952636 | 0.94697 |
|  | 53 | 0.957983 | 0.947231 |
|  | 54 | 0.95149 | 0.949843 |
|  | 55 | 0.959893 | 0.952194 |
|  | 56 | 0.958747 | 0.954284 |
|  | 57 | 0.95149 | 0.950104 |
|  | 58 | 0.953018 | 0.948276 |
|  | 59 | 0.957983 | 0.948798 |
|  | 60 | 0.947288 | 0.94697 |
|  | 61 | 0.958365 | 0.945925 |
|  | 62 | 0.952254 | 0.941223 |
|  | 63 | 0.961039 | 0.932341 |
|  | 64 | 0.954927 | 0.948798 |
|  | 65 | 0.949962 | 0.930773 |
|  | 66 | 0.950726 | 0.944619 |
|  | 67 | 0.956073 | 0.947231 |
|  | 68 | 0.952636 | 0.951933 |
|  | 69 | 0.9534 | 0.948798 |
|  | 70 | 0.950344 | 0.952456 |
|  | 71 | 0.952636 | 0.946708 |
|  | 72 | 0.957219 | 0.950627 |
|  | 73 | 0.948816 | 0.935998 |
|  | 74 | 0.958747 | 0.93861 |
|  | 75 | 0.951872 | 0.94488 |
|  | 76 | 0.956455 | 0.938871 |
|  | 77 | 0.954927 | 0.950627 |
|  | 78 | 0.960657 | 0.94906 |
|  | 79 | 0.954163 | 0.945925 |
|  | 80 | 0.954163 | 0.946186 |
|  | 81 | 0.954545 | 0.951149 |
|  | 82 | 0.951108 | 0.947492 |
|  | 83 | 0.956073 | 0.950888 |
|  | 84 | 0.956455 | 0.946186 |
|  | 85 | 0.960657 | 0.948537 |
|  | 86 | 0.954163 | 0.939133 |
|  | 87 | 0.958365 | 0.948015 |
|  | 88 | 0.953782 | 0.941484 |
|  | 89 | 0.956455 | 0.947753 |
|  | 90 | 0.956837 | 0.939133 |
|  | 91 | 0.954545 | 0.949582 |
|  | 92 | 0.955309 | 0.948537 |
|  | 93 | 0.9534 | 0.950888 |
|  | 94 | 0.959129 | 0.953239 |
|  | 95 | 0.952636 | 0.951149 |
|  | 96 | 0.958747 | 0.947753 |
|  | 97 | 0.952254 | 0.945925 |
|  | 98 | 0.948434 | 0.940961 |
|  | 99 | 0.957601 | 0.945402 |
|  | 100 | 0.962949 | 0.940178 |
