# Supplementary material for: Machine Learning–Based Survival Prediction Models for Young Patients With Gastric Cancer: Model Development and Validation Study
Source: JMIR Cancer. 2026 May 26;12:e86418. doi: 10.2196/86418 (PMC13211600; doi:10.2196/86418)

**Supplement file 3. Relative Feature Importance of Predictors in the Survival Prediction Model**

In the heatmap, colour intensity reflects the relative importance of the frequency with which each feature is selected, with darker shades indicating a higher frequency of selection.


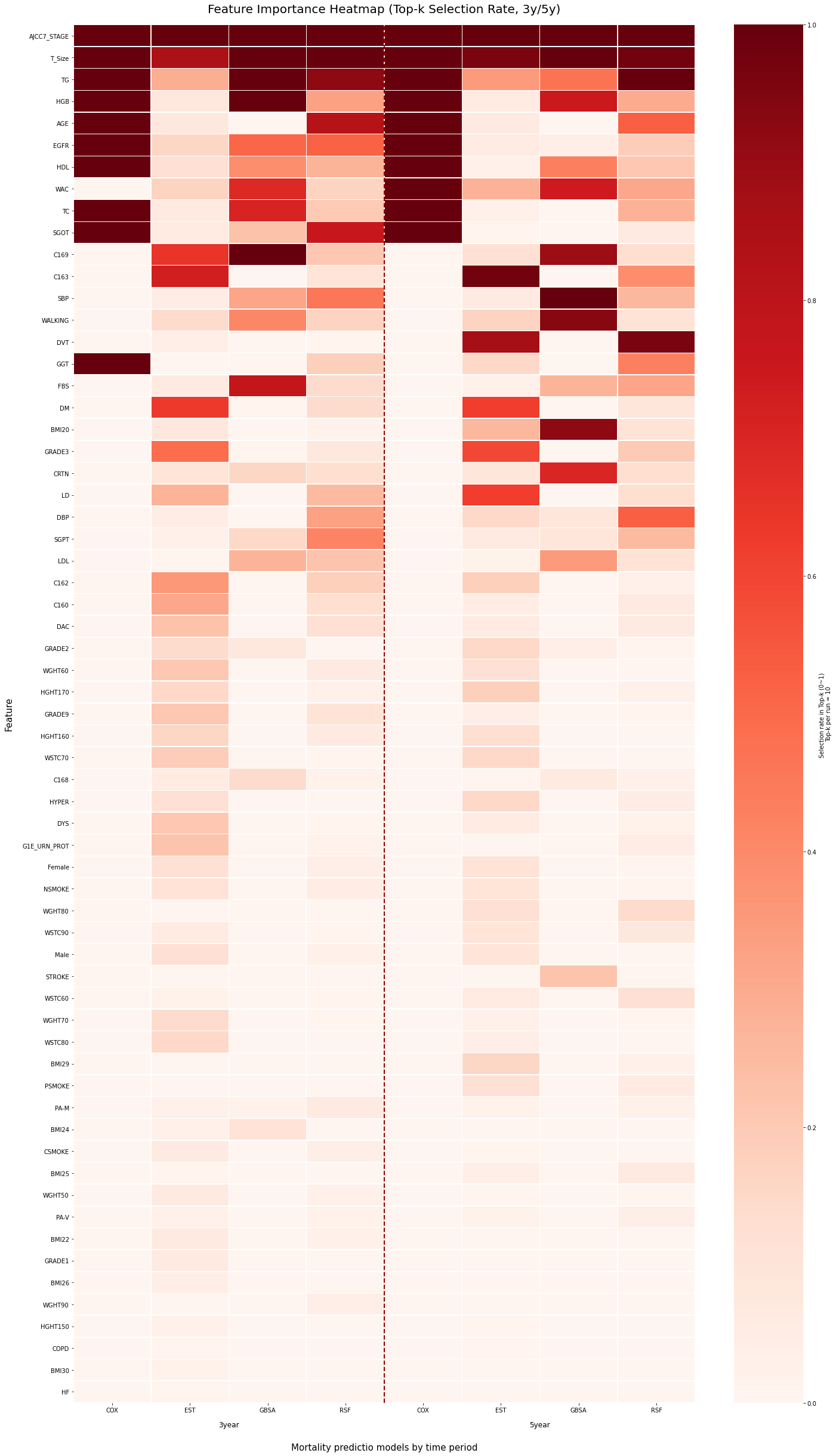

Supplement: Multimedia Appendix 3 [file cancer-v12-e86418-s003.docx]
