# Supplementary material for: Machine Learning–Based Survival Prediction Models for Young Patients With Gastric Cancer: Model Development and Validation Study
Source: JMIR Cancer. 2026 May 26;12:e86418. doi: 10.2196/86418 (PMC13211600; doi:10.2196/86418)
Supplement: Multimedia Appendix 4 [file cancer-v12-e86418-s004.docx]

**Supplement file 4. Univariate analysis results for all cause of death**

The tables present the univariate analysis of data related to young patients with gastric cancer for each time point, namely 3 years, and 5 years, respectively. Data is presented B, beta coefficient; HR(95% confidence interval) hazard ratio;

2.1 Univariate analysis result for all cause of death at 3 years

| **Variable** | **Β(Coefficient)** | **Hazard Ratio (HR)** | **P value** |
| --- | --- | --- | --- |
| Age (year) | -0.02513 | 0.975 | 0.3376 |
| T-size | 0.01812 | 1.018 | <0.0001 |
| BMI (kg/㎡) | -0.05026 | 0.951 | 0.2772 |
| Systolic blood pressure (mmHg) | 0.00181 | 1.002 | 0.8475 |
| Diastolic blood pressure (mmHg) | -0.00605 | 0.994 | 0.6459 |
| Haemoglobin level (g/dL) | -0.1804 | 0.835 | 0.0009 |
| Fasting blood sugar (mg/dL) | 0.00267 | 1.003 | 0.619 |
| Total cholesterol (mg/dL) | -0.00104 | 0.999 | 0.786 |
| Serum glutamic oxaloacetic transaminase (IU/L) | -0.02761 | 0.973 | 0.0817 |
| Serum glutamic pyruvic transaminase (IU/L) | -0.00993 | 0.990 | 0.2504 |
| Gamma glutamyl transpeptidase (IU/L) | -0.00357 | 0.996 | 0.286 |
| Triglycerides (mg/dL) | -0.00474 | 0.995 | 0.0196 |
| High-density lipoprotein (mg/dL) | -0.00003 | 1.000 | 0.9974 |
| Low-density lipoprotein (mg/dL) | 0.00168 | 1.002 | 0.567 |
| Serum creatine (mg/dL) | -0.17715 | 0.838 | 0.7359 |
| Estimated glomerular filtration rate (mL/min) | -0.00279 | 0.997 | 0.6789 |
| Weekly alcohol consumption (days) | -0.14992 | 0.861 | 0.135 |
| Daily alcohol consumption (glasses) | -0.04084 | 0.960 | 0.2099 |
| Vigorous physical activity (days in a week) | 0.00608 | 1.006 | 0.9396 |
| Moderate physical activity (days in a week) | 0.01764 | 1.018 | 0.8145 |
| Physical activity Walking (days in a week) | -0.02834 | 0.972 | 0.6115 |
| Female (%) | -0.12548 | 0.882 | 0.6323 |
| TCODE C160 (%) | -0.13309 | 0.875 | 0.8177 |
| TCODE C161 (%) | 0.04226 | 1.043 | 0.9587 |
| TCODE C162 (%) | -1.43697 | 0.238 | 0.0015 |
| TCODE C163 (%) | -1.96964 | 0.140 | 0.0001 |
| TCODE C164 (%) | -0.38961 | 0.677 | 0.7183 |
| TCODE C165 (%) | -14.23063 | 0.000 | 0.982 |
| TCODE C168 (%) | 0.48084 | 1.617 | 0.3876 |
| Morphology CODE 1 (%) | -0.000000005 | 1.000 | 1 |
| Morphology CODE 3 (%) | 13.01829 | 450581.300 | 0.9879 |
| GRADE 1 (%) | -1.03827 | 0.354 | 0.0916 |
| GRADE 2 (%) | -0.70419 | 0.495 | 0.1046 |
| GRADE 3 (%) | 0.05649 | 1.058 | 0.8441 |
| AJCC7 STAGE IA | -4.05644 | 0.017 | 0.0003 |
| AJCC7 STAGE IB | -16.40572 | 0.000 | 0.9832 |
| AJCC7 STAGE IIA | -2.24676 | 0.106 | 0.0666 |
| AJCC7 STAGE IIB | -1.98608 | 0.137 | 0.1049 |
| AJCC7 STAGE IIIA | -1.32296 | 0.266 | 0.252 |
| AJCC7 STAGE IIIB | 0.11445 | 1.121 | 0.9135 |
| AJCC7 STAGE IIIC | 0.12367 | 1.132 | 0.9052 |
| AJCC7 STAGE IV | 1.49154 | 4.444 | 0.144 |
| Height (cm)150 | 0.53066 | 1.700 | 0.4792 |
| Height (cm) 160 | 0.53468 | 1.707 | 0.4675 |
| Height (cm) 170 | 0.28532 | 1.330 | 0.7027 |
| Weight (kg, %) 40 | -0.33874 | 0.713 | 0.6136 |
| Weight (kg, %) 50 | -0.22779 | 0.796 | 0.6788 |
| Weight (kg, %) 60 | -0.31904 | 0.727 | 0.5638 |
| Weight (kg, %) 70 | -0.72722 | 0.483 | 0.219 |
| Weight (kg, %) 80 | -0.92871 | 0.395 | 0.224 |
| Waist circumference (cm, %) 60 | 0.31003 | 1.363 | 0.4416 |
| Waist circumference (cm, %) 70 | -0.18039 | 0.835 | 0.6374 |
| Waist circumference (cm, %) 80 | -0.27007 | 0.763 | 0.4905 |
| Protein in urine 1 negative (-) | 10.944 | 56613.410 | 0.9859 |
| Protein in urine 2 positive (±) | 10.74176 | 46247.150 | 0.9862 |
| Protein in urine 3 positive (+1) | 11.18509 | 72048.270 | 0.9856 |
| Protein in urine 4 positive (+2) | 13.01656 | 449798.600 | 0.9833 |
| Non-Smoker (%) | -0.00386 | 0.996 | 0.9891 |
| Past Smoker (%) | -0.48224 | 0.617 | 0.2692 |
| Atrial fibrillation (%) | -0.62334 | 0.536 | 0.3862 |
| Chronic kidney disease (%) | 13.0265 | 454295.600 | 0.9846 |
| Chronic obstructive pulmonary disease (%) | -0.02226 | 0.978 | 0.9588 |
| Diabetes (%) | 0.40355 | 1.497 | 0.1378 |
| Deep vein thrombosis | -1.33881 | 0.262 | 0.0019 |
| Dyslipidemia (%) | 0.26566 | 1.304 | 0.3643 |
| Heart failure (%) | -0.53784 | 0.584 | 0.2118 |
| Hypertension (%) | 0.21198 | 1.236 | 0.4998 |
| Liver disease (%) | -1.01317 | 0.363 | 0.0077 |
| Myocardial infarction (%) | 13.0265 | 454295.600 | 0.9846 |
| Obesity (%) | 11.00607 | 60238.740 | 0.983 |
| Stroke (%) | -0.65696 | 0.518 | 0.5147 |

2.2 Univariate analysis result for all cause of death at 5 years

| **Variable** | **Β(Coefficient)** | **Hazard Ratio (HR)** | **P value** |
| --- | --- | --- | --- |
| AGE (year) | -0.01174 | 0.988 | 0.6105 |
| T-SIZE | 0.01909 | 1.019 | 0.0001 |
| BMI (kg/㎡) | -0.04359 | 0.957 | 0.2658 |
| Systolic blood pressure (mmHg) | -0.00067 | 0.999 | 0.9337 |
| Diastolic blood pressure (mmHg) | -0.00473 | 0.995 | 0.6733 |
| Haemoglobin level (g/dL) | -0.16864 | 0.845 | 0.0005 |
| Fasting blood sugar (mg/dL) | 0.00217 | 1.002 | 0.6433 |
| Total cholesterol (mg/dL) | 0.00283 | 1.003 | 0.3722 |
| Serum glutamic oxaloacetic transaminase (IU/L) | -0.02111 | 0.979 | 0.0925 |
| Serum glutamic pyruvic transaminase (IU/L) | -0.00807 | 0.992 | 0.2504 |
| Gamma glutamyl transpeptidase (IU/L) | -0.00352 | 0.996 | 0.2139 |
| Triglycerides (mg/dL) | -0.00261 | 0.997 | 0.0811 |
| High-density lipoprotein (mg/dL) | -0.00174 | 0.998 | 0.8244 |
| Low-density lipoprotein (mg/dL) | 0.00302 | 1.003 | 0.1336 |
| Serum creatine (mg/dL) | -0.06803 | 0.934 | 0.8528 |
| Estimated glomerular filtration rate (mL/min) | -0.003 | 0.997 | 0.6025 |
| Weekly alcohol consumption (days) | -0.16674 | 0.846 | 0.055 |
| Daily alcohol consumption (glasses) | -0.04201 | 0.959 | 0.1314 |
| Vigorous physical activity (days in a week) | 0.0077 | 1.008 | 0.9101 |
| Moderate physical activity (days in a week) | 0.00627 | 1.006 | 0.9231 |
| Physical activity Walking (days in a week) | -0.00767 | 0.992 | 0.8704 |
| Female (%) | -0.03694 | 0.964 | 0.8697 |
| TCODE C160 (%) | 0.21055 | 1.234 | 0.6967 |
| TCODE C161 (%) | 0.07454 | 1.077 | 0.9273 |
| TCODE C162 (%) | -0.9951 | 0.37 | 0.0231 |
| TCODE C163 (%) | -1.72782 | 0.178 | 0.0004 |
| TCODE C164 (%) | -0.3658 | 0.694 | 0.7349 |
| TCODE C165 (%) | -1.31397 | 0.269 | 0.2238 |
| TCODE C168 (%) | 0.8309 | 2.295 | 0.1153 |
| Morphology CODE 1 (%) | -0.00006 | 1 | 1 |
| Morphology CODE 3 (%) | 13.01853 | 450686.8 | 0.9858 |
| GRADE 1 (%) | -1.29049 | 0.275 | 0.0336 |
| GRADE 2 (%) | -0.69631 | 0.498 | 0.0692 |
| GRADE 3 (%) | 0.24187 | 1.274 | 0.3239 |
| AJCC7 STAGE IA | -4.14358 | 0.016 | 0.0002 |
| AJCC7 STAGE IB | -2.06932 | 0.126 | 0.0732 |
| AJCC7 STAGE IIA | -1.61052 | 0.2 | 0.1498 |
| AJCC7 STAGE IIB | -1.1291 | 0.323 | 0.3028 |
| AJCC7 STAGE IIIA | -1.10192 | 0.332 | 0.3244 |
| AJCC7 STAGE IIIB | 0.36001 | 1.433 | 0.7304 |
| AJCC7 STAGE IIIC | 0.75585 | 2.129 | 0.46 |
| AJCC7 STAGE IV | 1.79265 | 6.005 | 0.0788 |
| Height (cm) 150 | 0.40088 | 1.493 | 0.5158 |
| Height (cm) 160 | 0.38927 | 1.476 | 0.5195 |
| Height (cm) 170 | 0.31035 | 1.364 | 0.6106 |
| Weight (kg, %) 40 | 0.01783 | 1.018 | 0.9745 |
| Weight (kg, %) 50 | -0.13782 | 0.871 | 0.7778 |
| Weight (kg, %) 60 | -0.49712 | 0.608 | 0.3227 |
| Weight (kg, %) 70 | -0.69731 | 0.498 | 0.1851 |
| Weight (kg, %) 80 | -0.06139 | 0.94 | 0.9124 |
| Waist circumference (cm, %) 60 | 0.3646 | 1.44 | 0.2858 |
| Waist circumference (cm, %) 70 | -0.20635 | 0.814 | 0.5307 |
| Waist circumference (cm, %) 80 | -0.26303 | 0.769 | 0.4321 |
| Protein in urine 1 negative (-) | -1.69234 | 0.184 | 0.0929 |
| Protein in urine 2 positive (±) | -1.80678 | 0.164 | 0.1178 |
| Protein in urine 3 positive (+1) | -1.04785 | 0.351 | 0.3923 |
| Protein in urine 4 positive (+2) | -0.59266 | 0.553 | 0.6752 |
| Non-Smoker (%) | 0.09694 | 1.102 | 0.6917 |
| Past-Smoker (%) | -0.38187 | 0.683 | 0.3023 |
| Atrial fibrillation (%) | -0.31231 | 0.732 | 0.6625 |
| Chronic kidney disease (%) | 0.31584 | 1.371 | 0.7535 |
| Chronic obstructive pulmonary disease (%) | 0.01097 | 1.011 | 0.9765 |
| Diabetes (%) | 0.24148 | 1.273 | 0.288 |
| Deep vein thrombosis (%) | -1.47642 | 0.228 | 0.001 |
| Dyslipidemia (%) | 0.20346 | 1.226 | 0.4223 |
| Heart failure (%) | -0.65463 | 0.52 | 0.0641 |
| Hypertension (%) | -0.1884 | 0.828 | 0.4388 |
| Liver disease (%) | -1.1355 | 0.321 | 0.0008 |
| Myocardial infarction (%) | 13.02712 | 454576.7 | 0.9818 |
| Obesity (%) | 11.00619 | 60245.82 | 0.9799 |
| Stroke (%) | -0.35838 | 0.699 | 0.7217 |
