# Supplementary material for: Machine Learning–Based Survival Prediction Models for Young Patients With Gastric Cancer: Model Development and Validation Study
Source: JMIR Cancer. 2026 May 26;12:e86418. doi: 10.2196/86418 (PMC13211600; doi:10.2196/86418)
Supplement: Multimedia Appendix 5 [file cancer-v12-e86418-s005.docx]

**Supplement file 5.** **Mapping between heatmap feature labels and clinical variables**

This supplementary file presents the mapping between the raw dataset variable names, the abbreviated feature labels used in the heatmap visualization of the machine learning models, and the corresponding clinical variables used in the manuscript tables.

| **Heatmap label** | **Table variable name** | **Description** |
| --- | --- | --- |
| AJCC7_STAGE | AJCC7 STAGE | AJCC 7th stage |
| T_Size | T-size | Tumor size |
| TG | Triglycerides (mg/dL) | Triglycerides (mg/dL) |
| HGB | Haemoglobin level (g/dL) | Haemoglobin level (g/dL) |
| AGE | Age (year) | Age (years) |
| EGFR | Estimated glomerular filtration rate (mL/min) | Estimated glomerular filtration rate |
| HDL | High-density lipoprotein (mg/dL) | High-density lipoprotein |
| WAC | Weekly alcohol consumption (days) | Weekly alcohol consumption |
| TC | Total cholesterol (mg/dL) | Total cholesterol |
| SGOT | Serum glutamic oxaloacetic transaminase (IU/L) | Serum glutamic oxaloacetic transaminase |
| C169 | TCODE C169 (%) | Topography CODE (%) |
| C163 | TCODE C163 (%) | Topography CODE (%) |
| SBP | Systolic blood pressure (mmHg) | Systolic blood pressure (mmHg) |
| WALKING | Physical activity Walking (days in a week) | Walking activity |
| DVT | Deep vein thrombosis (%) | Deep vein thrombosis |
| GGT | Gamma glutamyl transpeptidase (IU/L) | Gamma glutamyl transpeptidase |
| FBS | Fasting blood sugar (mg/dL) | Fasting blood sugar |
| DM | Diabetes (%) | Diabetes mellitus |
| BMI20 | BMI (kg/㎡) | Body mass index category |
| GRADE3 | GRADE 3 (%) | Tumor grade |
| CRTN | Serum creatine (mg/dL) | Serum creatinine |
| LD | Liver disease (%) | Liver disease |
| DBP | Diastolic blood pressure (mmHg) | Diastolic blood pressure |
| SGPT | Serum glutamic pyruvic transaminase (IU/L) | Serum glutamic pyruvic transaminase |
| LDL | Low-density lipoprotein (mg/dL) | Low-density lipoprotein |
| C162 | TCODE C162 (%) | Tumor topography C162 |
| C160 | TCODE C160 (%) | Tumor topography C160 |
| DAC | Daily alcohol consumption (glasses) | Daily alcohol consumption |
| GRADE2 | GRADE 2 (%) | Tumor grade |
| WGHT60 | Weight (kg, %) 60 | Weight category |
| HGHT170 | Height (cm) 170 | Height category |
| GRADE9 | GRADE 9 (%) | Tumor grade |
| HGHT160 | Height (cm) 160 | Height category |
| WSTC70 | Waist circumference (cm, %) 70 | Waist circumference category |
| C168 | TCODE C168 (%) | Tumor topography C168 |
| HYPER | Hypertension (%) | Hypertension |
| DYS | Dyslipidemia (%) | Dyslipidemia |
| G1E_URN_PROT | Protein in urine | Protein in urine |
| Female | Female (%) | Female |
| NSMOKE | Non-smoker (%) | Smoking status (%) |
| WGHT80 | Weight (kg, %) 80 | Weight category |
| WSTC90 | Waist circumference (cm, %) 90 | Waist circumference category |
| Male | Male (%) | Male |
| STROKE | Stroke (%) | Stroke |
| WSTC60 | Waist circumference (cm, %) 60 | Waist circumference category |
| WGHT70 | Weight (kg, %) 70 | Weight category |
| BMI29 | BMI (kg/㎡) | Body mass index category |
| PSMOKE | Past-Smoker (%) | Past smoker |
| PA M | Moderate physical activity (days in a week) | Moderate physical activity |
| BMI24 | BMI (kg/㎡) | Body mass index category |
| CSMOKE | Current smoker (%) | Current smoker |
| BMI25 | BMI (kg/㎡) | Body mass index category |
| WGHT50 | Weight (kg, %) 50 | Weight category |
| PA V | Vigorous physical activity (days in a week) | Vigorous physical activity |
| BMI22 | BMI (kg/㎡) | Body mass index category |
| GRADE1 | GRADE 1 (%) | Tumor grade |
| BMI26 | BMI (kg/㎡) | Body mass index category |
| WGHT90 | Weight (kg, %) 90 | Weight category |
| HGHT150 | Height (cm) 150 | Height category |
| COPD | Chronic obstructive pulmonary disease (%) | Chronic obstructive pulmonary disease |
| BMI30 | BMI (kg/㎡) | Body mass index category |
| HF | Heart failure (%) | Heart failure |
