# Supplementary material for: Machine Learning–Based Survival Prediction Models for Young Patients With Gastric Cancer: Model Development and Validation Study
Source: JMIR Cancer. 2026 May 26;12:e86418. doi: 10.2196/86418 (PMC13211600; doi:10.2196/86418)
Supplement: Multimedia Appendix 6 [file cancer-v12-e86418-s006.docx]

**Supplement file 6. Survival Curve for Younger and Older Patients**

The figure in question provides a comparison of the survival rates of patients in the young and old age groups within the entire population, as well as within the STAGE1, STAGE2, STAGE3 and STAGE4 groups. This is achieved by means of survival curves.

6.1. Comparisons survival curve between younger group and older group

| 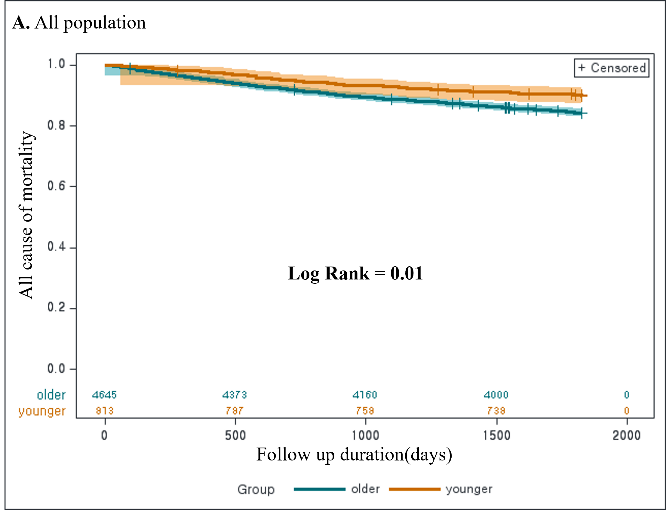 | 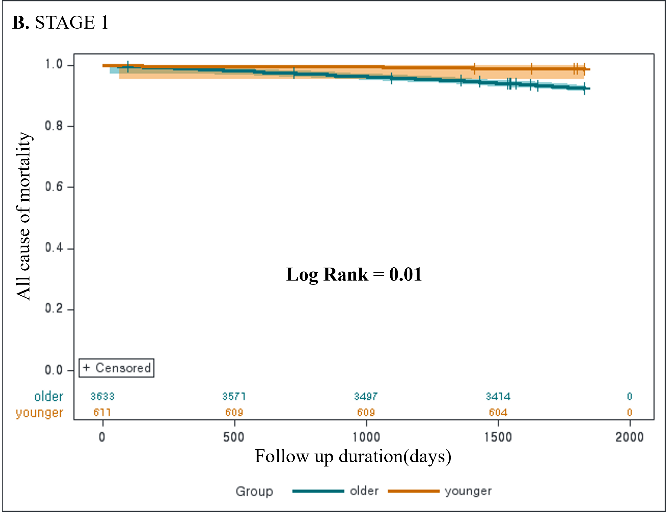 |
| --- | --- |
|  |  |
| 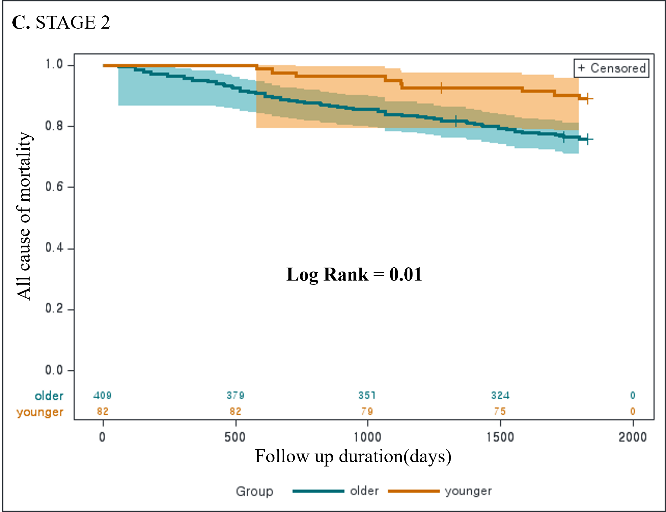 | 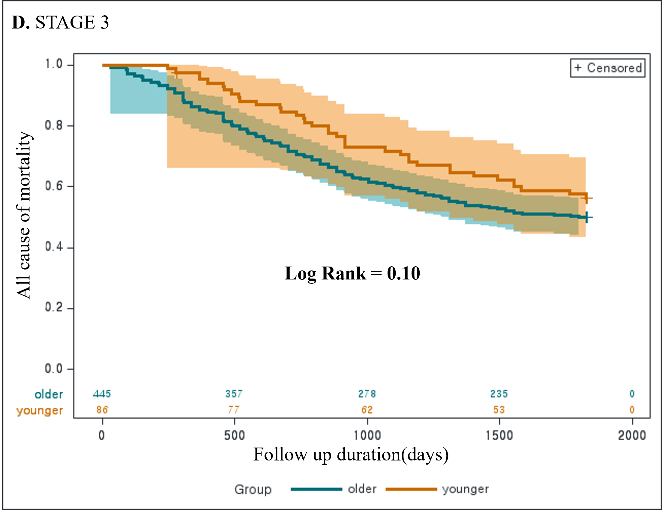 |
|  |  |
| 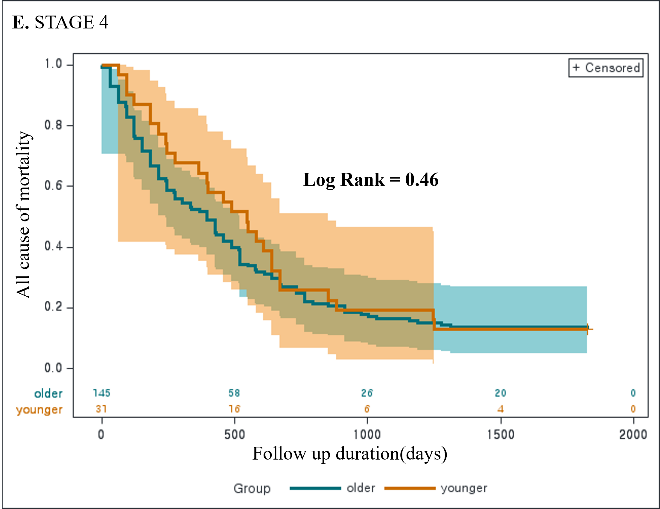 |  |
|  |  |
