# Supplementary material for: Machine Learning–Based Survival Prediction Models for Young Patients With Gastric Cancer: Model Development and Validation Study
Source: JMIR Cancer. 2026 May 26;12:e86418. doi: 10.2196/86418 (PMC13211600; doi:10.2196/86418)

**Supplement file 7. Survival Curve for STAGE and T-size**

This supplementary file presents Kaplan–Meier survival curves stratified by tumor stage and tumor size to illustrate differences in overall survival across risk groups.

7.1. Survival curves for Tumor-size


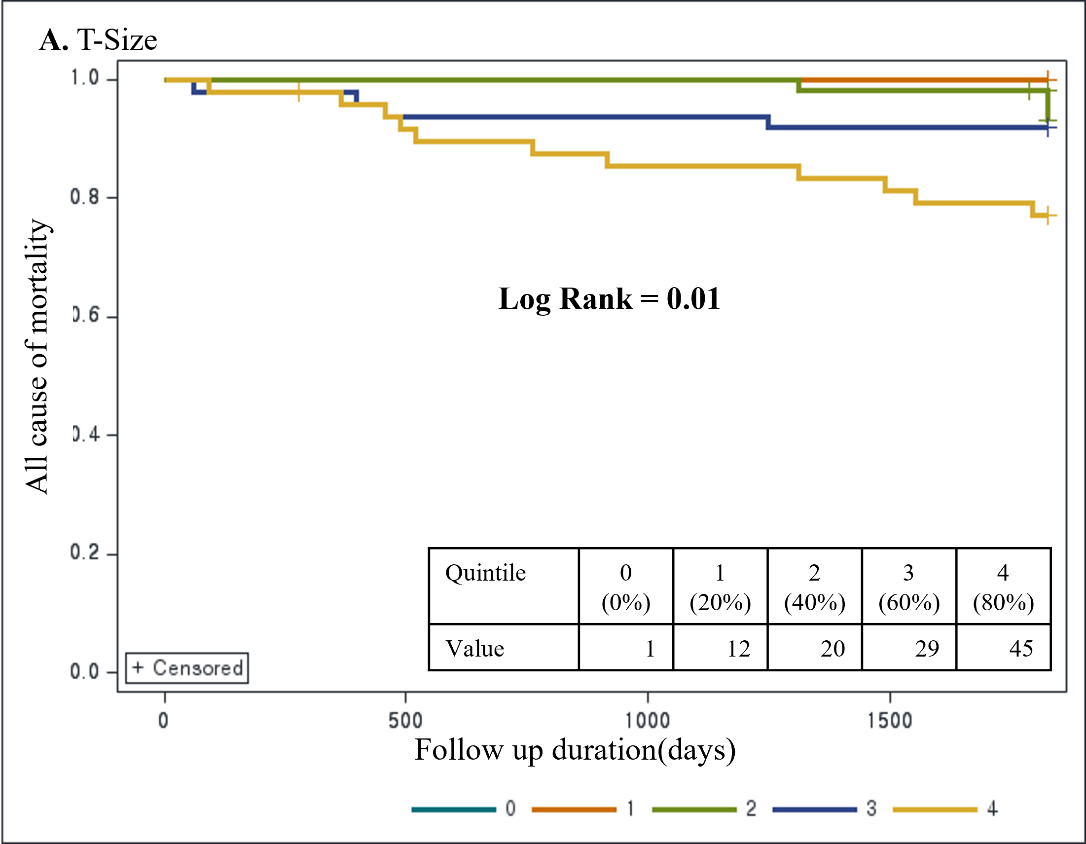


7.2 Survival curves for STAGE


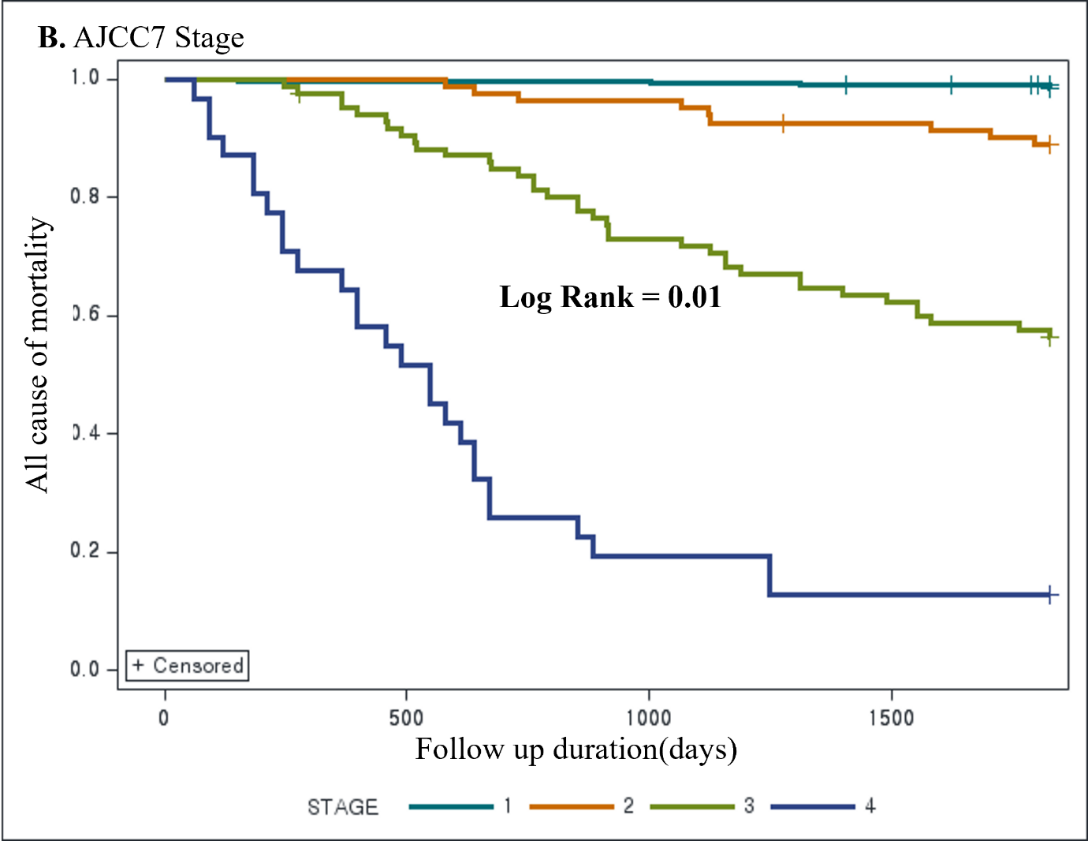

Supplement: Multimedia Appendix 7 [file cancer-v12-e86418-s007.docx]
