# Supplementary material for: Machine Learning–Based Survival Prediction Models for Young Patients With Gastric Cancer: Model Development and Validation Study
Source: JMIR Cancer. 2026 May 26;12:e86418. doi: 10.2196/86418 (PMC13211600; doi:10.2196/86418)
Supplement: Multimedia Appendix 8 [file cancer-v12-e86418-s008.docx]

**Supplement file 8. Comparsion of Predictive Performance Between AJCC Stage and Survival Machine Learning Model**

This supplementary table compares the predictive performance of the AJCC staging system and four survival prediction models for 1-, 3-, and 5-year mortality prediction. Model performance was assessed using the concordance index (C-index). For the machine learning models—Random Survival Forest (RSF), Gradient Boosting Survival Analysis (GBSA), and Extra Survival Trees (EST)—each algorithm was evaluated over 100 repeated runs, and the highest C-index achieved was reported to represent their optimal predictive performance. In contrast, the Cox proportional hazards model (CoxPH) and the AJCC staging system do not involve stochastic processes and therefore yield identical results across repeated analyses. Accordingly, these methods were evaluated once.

|  | 1-year prediction model | 3-year prediction model | 5-year prediction model |
| --- | --- | --- | --- |
| STAGE | **96.27** | 96.24 | 91.52 |
| RSF | 92.02 | **96.63** | 93.78 |
| GBSA | 81.05 | 95.41 | 90.21 |
| EST | 95.23 | 96.40 | **95.42** |
| CoxPH | 82.60 | 94.15 | 82.26 |
