# Supplementary material for: Machine Learning–Based Survival Prediction Models for Young Patients With Gastric Cancer: Model Development and Validation Study
Source: JMIR Cancer. 2026 May 26;12:e86418. doi: 10.2196/86418 (PMC13211600; doi:10.2196/86418)
Supplement: Multimedia Appendix 9 [file cancer-v12-e86418-s009.docx]

**Supplement file 9. Comparison of Year-Based Data Splitting and 7:3 Random Split**

9.1. Comparision performance evaluation between random splitting stratety(training: 70%, test:30%) and year-based splitting(training:2013-2014, test:2015)

| **Model** | **3-year** | **5-year** |
| --- | --- | --- |
|  | **C-index (95% CI)** | **C-index (95% CI)** |
| RSF | 82.13% (81.86-82.41) | 84.71% (84.54-84.87) |
| GBSA | 87.09% (87.06-87.11) | 87.29% (87.28-87.29) |
| EST | 86.87% (86.65-87.10) | 87.28% (87.11-87.45) |
| CoxPH | 75.52% | 70.50% |

C-index values for machine learning models represent the mean and 95% confidence intervals obtained from 100 repeated runs. The Cox model was evaluated once; therefore, confidence intervals are not reported.

9.2. Basic characteristics between random splitting strategy(training: 70%, test:30%) and year-based data splitting(training: 2013-2014, test: 2015)

| **Variables** | Random splitting strategy | | Year-based data splitting | |
| --- | --- | --- | --- | --- |
|  | Training (70%)  = 569 | Test (30%)  = 244 | Training  (2013-14)  = 583 | Test  (2015)  =230 |
| Age (year) | 44.42±4.78 | 43.98±4.61 | 44.30±4.73 | 44.26±4.75 |
| Female (%) | 40.77 | 41.39 | 40.14 | 43.04 |
| BMI (kg/㎡) | 23.26±2.96 | 23.47±3.04 | 23.37±2.97 | 23.22±3.03 |
| Height (cm) |  |  |  |  |
| 150 ≤ H <160 | 25.83 | 21.72 | 24.53 | 24.78 |
| 160 ≤ H <170 | 36.73 | 37.70 | 35.51 | 40.87 |
| 170 ≤ H < 180 | 32.51 | 34.84 | 34.65 | 29.57 |
| 180 ≤ H | 4.92 | 5.74 | 5.32 | 4.78 |
| Weight (kg, %) |  |  |  |  |
| 40 ≤ W < 50 | 8.79 | 8.79 | 6.69 | 11.3 |
| 50 ≤ W < 60 | 28.47 | 28.47 | 27.1 | 27.39 |
| 60 ≤ W < 70 | 26.71 | 31.97 | 30.02 | 23.91 |
| 70 ≤ W < 80 | 23.02 | 24.18 | 24.01 | 21.74 |
| 80 ≤ W < 90 | 9.49 | 6.15 | 7.2 | 11.74 |
| 90 ≤ W | 3.51 | 7.38 | 4.97 | 3.91 |
| Waist circumference (cm, %) |  |  |  |  |
| 60 ≤ WC < 70 | 17.93 | 12.3 | 15.09 | 19.13 |
| 70 ≤ WC < 90 | 31.63 | 38.11 | 32.42 | 36.52 |
| 80 ≤ WC < 90 | 33.39 | 31.56 | 35.33 | 26.52 |
| 90 ≤ WC | 17.05 | 18.03 | 17.15 | 17.83 |
| Systolic blood pressure (mmHg) | 119.01±13.55 | 119.51±14.18 | 118.98±13.74 | 119.60±13.72 |
| Diastolic blood pressure (mmHg) | 75.01±9.67 | 75.37±10.72 | 75.05±10.15 | 75.29±9.60 |
| Haemoglobin level (g/dL) | 13.98±1.98 | 14.04±1.90 | 14.01±2.04 | 13.97±1.73 |
| Fasting blood sugar (mg/dL) | 99.14±23.43 | 96.93±18.14 | 98.71±22.63 | 97.89±20.32 |
| Total cholesterol (mg/dL) | 192.92±35.34 | 191.34±33.55 | 192.87±34.73 | 191.36±35.02 |
| Serum glutamic oxaloacetic transaminase (IU/L) | 25.60±13.92 | 24.90±15.64 | 25.49±15.50 | 25.13±11.42 |
| Serum glutamic pyruvic transaminase (IU/L) | 26.50±21.89 | 23.71±17.40± | 26.03±21.68 | 24.73±17.88 |
| Gamma glutamyl transpeptidase (IU/L) | 45.26±64.33 | 39.32±64.31 | 45.13±68.95 | 39.31±50.71 |
| Triglycerides (mg/dL) | 139.69±111.45 | 129.38±81.08 | 137.26±107.35 | 134.93±92.58 |
| High-density lipoprotein (mg/dL) | 53.46±14.51 | 52.90±13.59 | 52.81±13.92 | 54.52±14.95 |
| Low-density lipoprotein (mg/dL) | 113.061±40.00 | 112.80±30.78 | 113.92±39.18 | 110.60±32.62 |
| Serum creatine (mg/dL) | 0.86±0.18 | 0.92±0.57 | 0.89±0.38 | 0.86±0.25 |
| Estimated glomerular filtration rate (mL/min) | 92.50±19.59 | 90.73±19.91 | 91.31±18.84 | 93.63±21.65 |
| Protein in urine (%) |  |  |  |  |
| 1 negative (-) | 92.79 | 95.49 | 93.83 | 93.04 |
| 2 positive (±) | 4.75 | 2.87 | 4.12 | 4.35 |
| 3 positive (+1) | 1.58 | *** | 1.2 | *** |
| 4 positive (+2) | *** | *** | *** | *** |
| 5 positive (+3) | *** | *** | *** | *** |
| 6 positive (+4) | *** | *** | *** | *** |
| Topography CODE (%) |  |  |  |  |
| C160 | 3.87 | 2.46 | 3.6 | 3.04 |
| C161 | 1.05 | *** | *** | *** |
| C162 | 52.02 | 48.36 | 50.43 | 52.17 |
| C163 | 34.62 | 40.98 | 37.05 | 35.22 |
| C164 | *** | *** | *** | *** |
| C165 | 2.11 | *** | *** | *** |
| C166 | *** | *** | 2.06 | *** |
| C168 | 2.99 | *** | 2.4 | *** |
| C169 | 2.81 | 4.51 | 2.92 | 4.35 |
| Morphology CODE (%) |  |  |  |  |
| 1. Squamous and transitional cell carcinoma (8051– 8084, 8120–8131)) | *** | *** | *** | *** |
| 3. Adenocarcinoma (8140–8149, 8160–8163, 8190–8221, 8260–8337, 8350–8552, 8570–8576, 8940–8941) | 99.12 | 98.36 | 98.8 | 99.13 |
| 4. Other specific carcinomas (8030–8046, 8150–8157, 8170–8180, 8230–8255, 8340–8347, 8560–8562, 8580–8671) | *** | *** | *** | *** |
| 5. Unspecified carcinomas (NOS) (8010–8015, 8020–8022, 8050) | *** | *** | 1.03 | *** |
| 17. Unspecified types of cancer (8000–8005) | *** | *** | *** | *** |
| GRADE (%) |  |  |  |  |
| 1. Well-differentiated, differentiated, NOS | 10.90 | 13.93 | 11.32 | 13.04 |
| 2. Moderately differentiated, moderately well differentiated, intermediate differentiation | 19.33 | 20.90 | 20.41 | 18.26 |
| 3. Poorly differentiated; dedifferentiated | 37.61 | 34.43 | 37.74 | 33.91 |
| 4. Undifferentiated, anaplastic | *** | *** | *** | *** |
| 9. Differentiation unknown, not stated, or not applicable | 32.16 | 30.74 | 30.53 | 34.78 |
| AJCC7 STAGE (%) |  |  |  |  |
| IA | 68.19 | 69.67 | 67.41 | 71.74 |
| IB | 7.03 | 5.33 | 7.38 | 4.35 |
| IIA | 4.75 | 7.79 | 6.00 | 4.78 |
| IIB | 4.39 | 4.51 | 4.29 | 4.78 |
| IIIA | 4.22 | 2.05 | 3.43 | 3.91 |
| IIIB | 2.28 | 4.51 | 3.43 | *** |
| IIIC | 4.22 | 3.69 | 3.77 | 4.78 |
| IIIN | *** | *** | *** | *** |
| IV | 4.39 | 2.46 | 3.77 | *** |
| Unknown | *** | *** | *** | *** |
| T-size | 31.81±29.94 | 30.40±25.75 | 31.83±28.52 | 30.26±29.31 |
| Atrial fibrillation (%) | 1.23 | 3.28 | 1.72 | ** |
| Chronic kidney disease (%) | 1.58 | *** | 1.20 | 2.61 |
| Chronic obstructive pulmonary disease (%) | 10.19 | 9.84 | 10.63 | 8.70 |
| Diabetes (%) | 46.40 | 40.98 | 44.08 | 46.52 |
| Deep vein thrombosis | 2.99 | 3.28 | 2.92 | 3.48 |
| Dyslipidaemia (%) | 78.56 | 75.41 | 78.22 | 76.09 |
| Heart failure (%) | 5.45 | 7.79 | 5.83 | 6.96 |
| Hypertension (%) | 25.83 | 25.41 | 25.04 | 27.39 |
| Liver disease (%) | 71.88 | 66.80 | 70.33 | 70.43 |
| Myocardial infarction (%) | 1.76 | *** | 1.54 | *** |
| Obesity(%) | *** | *** | *** | *** |
| Stroke (%) | *** | *** | *** | *** |
| Smoking status (%) |  |  |  |  |
| Non-smoker | 49.74 | 47.13 | 48.37 | 50.43 |
| Past smoker | 16.52 | 20.49 | 17.32 | 18.7 |
| Current smoker | 33.74 | 32.38 | 34.31 | 30.87 |
| Weekly alcohol consumption (days) | 1.28±1.50 | 1.27±1.50 | 1.31±1.53 | 1.21±1.40 |
| Daily alcohol consumption (glasses) | 3.89±4.46 | 3.71±4.66 | 3.83±4.48 | 3.84±4.64 |
| Vigorous physical activity  (days in a week) | 1.07±1.62 | 0.98±1.56 | 0.98±1.52 | 1.21±1.80 |
| Moderate physical activity  (days in a week) | 1.22±1.65 | 1.31±1.78 | 1.19±1.66 | 1.39±1.77 |
| Physical activity Walking  (days in a week) | 2.52±2.32 | 2.90±2.47 | 2.58±2.34 | 2.76±2.44 |
| 3 year survival time (days) | 1051.44±  167.88 | 1066.20±  146.61 | 1054.23±  167.30 | 1060.03±147.35 |
| 5 year survival time (days) | 1705.74±  365.74 | 1754.23±  298.93 | 1714.55±  355.83 | 1734.86±326.00 |
| All cause of death for 3 year (%) | 8.44 | 4.51 | 6.96 | 7.38 |
| All cause of death for 5 year (%) | 11.25 | 6.97 | 10.46 | 8.70 |

*** count less than or equal to 5
